# Non‐pharmacological interventions for prophylaxis of vestibular migraine

**DOI:** 10.1002/14651858.CD015321.pub2

**Published:** 2023-04-12

**Authors:** Katie E Webster, Afrose Dor, Kevin Galbraith, Luma Haj Kassem, Natasha A Harrington-Benton, Owen Judd, Diego Kaski, Otto R Maarsingh, Samuel MacKeith, Jaydip Ray, Vincent A Van Vugt, Martin J Burton

**Affiliations:** Cochrane ENT, Nuffield Department of Surgical SciencesUniversity of OxfordOxfordUK; Wadham CollegeUniversity of OxfordOxfordUK; Cochrane ENTNuffield Department of Surgical Sciences, University of OxfordOxfordUK; Aleppo University HospitalAleppoSyrian Arab Republic; Ménière's SocietyDorkingUK; ENT DepartmentUniversity Hospitals of Derby and Burton NHS Foundation TrustDerbyUK; National Hospital for Neurology and NeurosurgeryLondonUK; Amsterdam UMC, Vrije Universiteit Amsterdam, Department of General PracticeAmsterdam Public Health Research InstituteAmsterdamNetherlands; ENT DepartmentOxford University Hospitals NHS Foundation TrustOxfordUK; University of SheffieldSheffieldUK; Nuffield Department of Surgical SciencesUniversity of OxfordOxfordUK

**Keywords:** Adult, Female, Humans, Male, Cognitive Behavioral Therapy, Headache, Migraine Disorders, Migraine Disorders/prevention & control, Vertigo

## Abstract

**Background:**

Vestibular migraine is a form of migraine where one of the main features is recurrent attacks of vertigo. These episodes are often associated with other features of migraine, including headache and sensitivity to light or sound. These unpredictable and severe attacks of vertigo can lead to a considerable reduction in quality of life. The condition is estimated to affect just under 1% of the population, although many people remain undiagnosed. A number of interventions have been used, or proposed to be used, as prophylaxis for this condition, to help reduce the frequency of the attacks. Many of these interventions include dietary, lifestyle or behavioural changes, rather than medication.

**Objectives:**

To assess the benefits and harms of non‐pharmacological treatments used for prophylaxis of vestibular migraine.

**Search methods:**

The Cochrane ENT Information Specialist searched the Cochrane ENT Register; Central Register of Controlled Trials (CENTRAL); Ovid MEDLINE; Ovid Embase; Web of Science; ClinicalTrials.gov; ICTRP and additional sources for published and unpublished trials. The date of the search was 23 September 2022.

**Selection criteria:**

We included randomised controlled trials (RCTs) and quasi‐RCTs in adults with definite or probable vestibular migraine comparing dietary modifications, sleep improvement techniques, vitamin and mineral supplements, herbal supplements, talking therapies, mind‐body interventions or vestibular rehabilitation with either placebo or no treatment. We excluded studies with a cross‐over design, unless data from the first phase of the study could be identified.

**Data collection and analysis:**

We used standard Cochrane methods. Our primary outcomes were: 1) improvement in vertigo (assessed as a dichotomous outcome ‐ improved or not improved), 2) change in vertigo (assessed as a continuous outcome, with a score on a numerical scale) and 3) serious adverse events. Our secondary outcomes were: 4) disease‐specific health‐related quality of life, 5) improvement in headache, 6) improvement in other migrainous symptoms and 7) other adverse effects. We considered outcomes reported at three time points: < 3 months, 3 to < 6 months, > 6 to 12 months. We used GRADE to assess the certainty of evidence for each outcome.

**Main results:**

We included three studies in this review with a total of 319 participants. Each study addressed a different comparison and these are outlined below. We did not identify any evidence for the remaining comparisons of interest in this review.

***Dietary interventions (probiotics) versus placebo***

We identified one study with 218 participants (85% female). The use of a probiotic supplement was compared to a placebo and participants were followed up for two years. Some data were reported on the change in vertigo frequency and severity over the duration of the study. However, there were no data regarding improvement of vertigo or serious adverse events.

***Cognitive behavioural therapy (CBT) versus no intervention***

One study compared CBT to no treatment in 61 participants (72% female). Participants were followed up for eight weeks. Data were reported on the change in vertigo over the course of the study, but no information was reported on the proportion of people whose vertigo improved, or on the occurrence of serious adverse events.

***Vestibular rehabilitation versus no intervention***

The third study compared the use of vestibular rehabilitation to no treatment in a group of 40 participants (90% female) and participants were followed up for six months. Again, this study reported some data on change in the frequency of vertigo during the study, but no information on the proportion of participants who experienced an improvement in vertigo or the number who experienced serious adverse events.

We are unable to draw meaningful conclusions from the numerical results of these studies, as the data for each comparison of interest come from single, small studies and the certainty of the evidence was low or very low.

**Authors' conclusions:**

There is a paucity of evidence for non‐pharmacological interventions that may be used for prophylaxis of vestibular migraine. Only a limited number of interventions have been assessed by comparing them to no intervention or a placebo treatment, and the evidence from these studies is all of low or very low certainty. We are therefore unsure whether any of these interventions may be effective at reducing the symptoms of vestibular migraine and we are also unsure whether they have the potential to cause harm.

## Plain language summary

**How effective are non‐drug treatments used to prevent attacks of vestibular migraine?**

**Key messages**

It is not clear whether any of these treatments are effective at preventing attacks of vestibular migraine.

Few studies have assessed the possible benefits and harms of using these treatments to prevent attacks.

The studies found are small and the results are inconclusive.

**What is vestibular migraine?**

Migraine (sometimes known as 'headache migraine') is a common condition that causes recurrent headaches. Vestibular migraine is a related condition where the main symptoms are recurring episodes of severe dizziness or vertigo (a spinning sensation). These episodes are often associated with headache, or other migraine‐like symptoms (such as sensitivity to light or sound, nausea or vomiting). It is a relatively common condition, which affects up to 1 in every 100 people, and can have severe effects on day‐to‐day life.

**How is vestibular migraine treated?**

Typical treatment plans include medications to try and stop an attack of vertigo once it has started, or to improve the symptoms. In addition, people may use treatments intended to prevent attacks from starting (prophylactic or preventative treatment). There are no widely recommended treatments to prevent or manage the symptoms of a vestibular migraine attack. People are sometimes advised to take medications used to treat headache migraine. The assumption is that these medicines may also work for vestibular migraine. Sometimes people may also try non‐drug treatments to help prevent or improve their symptoms and try to reduce how often attacks occur. This might include changing their diet (such as eating less salt) or taking supplements (such as vitamins or minerals). Other treatments range from talking therapies to physiotherapy.

**What did we want to find out?**

We wanted to find out:

‐ whether there was evidence that any non‐drug treatments work to prevent attacks of vestibular migraine, or reduce the symptoms when an attack occurs;

‐ whether the treatments might cause any harm.

**What did we do?**

We searched for studies including adults that compared different types of treatment to either no treatment or placebo (dummy) treatment. We used standard methods to assess the quality of the evidence. We rated our confidence in the evidence, based on factors such as study methods, the number of participants in them and the consistency of findings across studies.

**What did we find?**

We found three studies, which included a total of 319 people (84% were female). These studies looked at three different types of treatments, to assess whether they might help to prevent vestibular migraine attacks, or help to reduce the symptoms when episodes occur.

*Probiotics*

The first study looked at the use of probiotics ‐ these are a type of bacteria that can be taken as a supplement to the normal diet. It was unclear whether this treatment made any difference to vertigo symptoms in people with vestibular migraine, or changed the frequency of attacks. The study did not report any information on possible harms, so we do not know if there are any risks associated with taking this treatment.

*Cognitive behavioural therapy (CBT)*

CBT is a type of talking therapy, which aims to help improve problems by changing the way people think and behave. One small study assessed whether CBT helps to prevent attacks of vestibular migraine. However, it was not clear whether this treatment had an effect on people's symptoms or the frequency of vertigo attacks. Again, no information was reported on possible harms of the treatment.

*Vestibular rehabilitation*

Vestibular rehabilitation is a form of physiotherapy that includes specific exercises to try and improve problems with balance. Another small study compared vestibular rehabilitation to no treatment in people with vestibular migraine. Again, the results were inconclusive and we cannot be sure whether this treatment has any effect on changing people's symptoms, or the frequency of attacks. No information was reported on potential harms of the treatment.

**What are the limitations of the evidence?**

We have very little confidence in the evidence included in this review. The three studies conducted were small and there were some problems with their conduct, which means that the results may be unreliable. Overall, it was not clear whether any of the treatments assessed were of benefit in the treatment of vestibular migraine. Importantly, none of the studies reported any information about harms, so we cannot tell if there are any possible risks associated with these treatments.

**How up‐to‐date is this evidence?**

This evidence is up‐to‐date to September 2022.

## Summary of findings

**Summary of findings 1:** Dietary intervention (probiotics) compared to placebo for prophylaxis of vestibular migraine

**Dietary intervention (probiotics) compared to placebo for prophylaxis of vestibular migraine**						
**Patient or population:** adults with vestibular migraine **Setting:** outpatient **Intervention:** probiotics **Comparison:** placebo						
**Outcomes**	Anticipated absolute effects^*^ (95% CI)		**Relative effect (95% CI)**	**№ of participants (studies)**	**Certainty of the evidence (GRADE)**	**Comments**
	**Risk with placebo**	**Risk with probiotics**				
Improvement in vertigo	This outcome was not reported.					
Change in vertigo: global score Assessed with: 10‐point Likert scale; higher scores = worse symptoms Scale from: 1 to 10 Follow‐up: range 3 months to 6 months	The mean change in vertigo global score was ‐2.6 points	MD 2.2 points lower (3.73 lower to 0.67 lower)	—	204 (1 RCT)	⊕⊝⊝⊝ Very low^1,2,3,4^	Probiotics may result in a reduction (improvement) in the change in vertigo when assessed with a global score at 3 to 6 months, but the evidence is very uncertain.
Change in vertigo: frequency Assessed with: number of attacks per week Follow‐up: range 3 months to 6 months	The mean change in vertigo frequency was ‐0.9 attacks per week	MD 0.7 attacks per week lower (2.39 lower to 0.99 higher)	—	204 (1 RCT)	⊕⊝⊝⊝ Very low^1,2,3,5^	The evidence is very uncertain about the effect of probiotics on vertigo frequency at 3 to 6 months.
Serious adverse events Follow‐up: range 3 months to 6 months	No studies reported this outcome.					
*The risk in the intervention group (and its 95% confidence interval) is based on the assumed risk in the comparison group and the **relative effect** of the intervention (and its 95% CI). **CI:** confidence interval; **MD:** mean difference; **RCT:** randomised controlled trial						
**GRADE Working Group grades of evidence** **High certainty:** we are very confident that the true effect lies close to that of the estimate of the effect. **Moderate certainty:** we are moderately confident in the effect estimate: the true effect is likely to be close to the estimate of the effect, but there is a possibility that it is substantially different. **Low certainty:** our confidence in the effect estimate is limited: the true effect may be substantially different from the estimate of the effect. **Very low certainty:** we have very little confidence in the effect estimate: the true effect is likely to be substantially different from the estimate of effect.						

^1^High risk of reporting bias with this outcome, as vertigo results are reported differently to the process specified in the methods section of the study. ^2^Concerns over lack of detail on randomisation methods, potential for variable use of additional interventions in each study group over the study period (2 years), discrepancy in trial data reporting and indication of a per protocol analysis. ^3^Sample size fails to meet the optimal information size (OIS) for this outcome, taken to be < 400 participants for a continuous outcome or < 300 events for a dichotomous outcome. ^4^Unclear description of the scale used to measure this outcome and whether it is considering vertigo symptoms only, or also quality of life. ^5^Confidence interval includes the possibility of potential benefit, as well as possible harm from the intervention. Minimally important difference assumed to be approximately 1 attack per week.

**Summary of findings 2:** Cognitive behavioural therapy (CBT) compared to no treatment for prophylaxis of vestibular migraine

**Cognitive behavioural therapy (CBT) compared to no treatment for prophylaxis of vestibular migraine**						
**Patient or population:** adults with vestibular migraine **Setting:** outpatient **Intervention:** cognitive behavioural therapy (CBT) **Comparison:** no treatment						
**Outcomes**	Anticipated absolute effects^*^ (95% CI)		**Relative effect (95% CI)**	**№ of participants (studies)**	**Certainty of the evidence (GRADE)**	**Comments**
	**Risk with no treatment**	**Risk with CBT**				
Improvement in vertigo	This outcome was not reported.					
Vertigo (global score) at < 3 months Assessed with: VSS‐SF (higher scores = worse symptoms) Scale from: 0 to 60	The mean VSS‐SF score in the control group was 13.89 points	MD 1.23 points higher (7.41 lower to 9.87 higher)	—	34 (1 RCT)	⊕⊝⊝⊝ Very low^1,2,3,4^	The evidence is very uncertain about the effect of CBT on vertigo at < 3 months.
Serious adverse events	This outcome was not reported.					
*The risk in the intervention group (and its 95% confidence interval) is based on the assumed risk in the comparison group and the **relative effect** of the intervention (and its 95% CI). **CI:** confidence interval; **MD:** mean difference; **RCT:** randomised controlled trial; **VSS‐SF:** Vertigo Symptom Scale Short Form						
**GRADE Working Group grades of evidence** **High certainty:** we are very confident that the true effect lies close to that of the estimate of the effect. **Moderate certainty:** we are moderately confident in the effect estimate: the true effect is likely to be close to the estimate of the effect, but there is a possibility that it is substantially different. **Low certainty:** our confidence in the effect estimate is limited: the true effect may be substantially different from the estimate of the effect. **Very low certainty:** we have very little confidence in the effect estimate: the true effect is likely to be substantially different from the estimate of effect.						

^1^High risk of performance and detection bias, due to lack of blinding. High risk of attrition bias due to missing outcome data. ^2^VSS‐SF includes other symptoms (such as autonomic and anxiety symptoms) and does not specifically relate to vertigo symptoms. No data were available for the vestibular‐balance subscale. ^3^Extremely small sample size. ^4^Confidence intervals include the possibility of either benefit or harm from the intervention.

**Summary of findings 3:** Vestibular rehabilitation compared to no treatment for prophylaxis of vestibular migraine

**Vestibular rehabilitation compared to no treatment for prophylaxis of vestibular migraine**						
**Patient or population:** adults with vestibular migraine **Setting:** outpatient **Intervention:** vestibular rehabilitation **Comparison:** no treatment						
**Outcomes**	Anticipated absolute effects^*^ (95% CI)		**Relative effect (95% CI)**	**№ of participants (studies)**	**Certainty of the evidence (GRADE)**	**Comments**
	**Risk with no treatment**	**Risk with vestibular rehabilitation**				
Improvement in vertigo	This outcome was not reported.					
Change in vertigo Assessed with: median number of attacks per month Follow‐up: range 3 months to 6 months	The median number of vertigo attacks per month was 4.5 in the control group	The median number of vertigo attacks per month was 1.5 in the vestibular rehabilitation group. No statistical analysis was presented.	—	40 (1 RCT)	⊕⊝⊝⊝ Very low^1,2,3^	The evidence is very uncertain as to whether vestibular rehabilitation affects the number of vertigo attacks at 3 to 6 months.
Serious adverse effects	This outcome was not reported.					
*The risk in the intervention group (and its 95% confidence interval) is based on the assumed risk in the comparison group and the **relative effect** of the intervention (and its 95% CI). **CI:** confidence interval; **RCT:** randomised controlled trial						
**GRADE Working Group grades of evidence** **High certainty:** we are very confident that the true effect lies close to that of the estimate of the effect. **Moderate certainty:** we are moderately confident in the effect estimate: the true effect is likely to be close to the estimate of the effect, but there is a possibility that it is substantially different. **Low certainty:** our confidence in the effect estimate is limited: the true effect may be substantially different from the estimate of the effect. **Very low certainty:** we have very little confidence in the effect estimate: the true effect is likely to be substantially different from the estimate of effect.						

^1^Very serious risk of bias due to quasi‐randomised allocation, lack of blinding of study participants and outcome assessors, and attrition bias. ^2^Use of background pharmacological therapy in both groups ‐ details on the nature of this treatment are not provided. ^3^Very small study. Unable to appropriately compare groups with the data presented.

## Background

### Description of the condition

Vestibular migraine is a form of migraine in which a prominent symptom, often *the* predominant symptom, is recurrent attacks of vertigo (Dieterich 1999; Lempert 2009). These episodes of vertigo are associated with other headache migraine features, such as headache or sensitivity to light or sound. 

The diagnosis of vestibular migraine is challenging because of the overlap of some symptoms with both other balance disorders (such as Ménière's disease) and with headache migraine. People suffering from headache migraine may experience occasional vestibular symptoms, but this does not amount to a diagnosis of 'vestibular migraine'. 

There is now an agreed international classification system that includes categories for 'definite' and 'probable' vestibular migraine (Lempert 2012; described in Appendix 1). In brief, a definite diagnosis of vestibular migraine requires at least five episodes of vestibular symptoms (of moderate to severe intensity) lasting between five minutes and 72 hours. At least half of the episodes must be associated with migrainous features (such as headache, photophobia, phonophobia or a visual aura) and individuals must also have a history of migraine. A diagnosis of 'probable' vestibular migraine requires similar features, but individuals have either migrainous features or a history of migraines (both are not required). Prior to this internationally agreed classification, the criteria proposed by Neuhauser and colleagues were widely used to identify people with vestibular migraine (Neuhauser 2001). There is a great deal of similarity between these classification systems, although the Neuhauser criteria do not require a certain number of episodes, or duration of episodes, to make the diagnosis.

Vestibular migraine is the most common cause of recurrent spontaneous vertigo in adults (Dieterich 2016). The lifetime prevalence of vestibular migraine has been estimated at just under 1% (Neuhauser 2006) and, as such, it is much more common than Ménière's disease. A significant number of cases may still go undiagnosed because of unfamiliarity with the condition or the diagnostic criteria. The disorder may have a slight female preponderance (Lempert 2009). As with many migraine disorders, a genetic susceptibility has been described and candidate genes have been suggested (Frejo 2016).

The pathophysiology of vestibular migraine is still uncertain, but it seems likely to involve similar mechanisms to those of headache migraine. These include activation of the trigeminovascular system (TGVS), which receives nociceptive signals from the large intracranial vessels and the dura (Bernstein 2012). Activation of the TGVS results in neuronal stimulation within parts of the brain involved in pain perception and sensory processing (including the thalamus and the periaqueductal grey) and also causes the release of vasoactive neuropeptides, such as calcitonin gene‐related peptide (CGRP). These, in turn, cause dilatation of the meningeal vessels, extravasation of fluid from the vasculature and release of other inflammatory substances in the dura (Pietrobon 2003), creating a cycle of nerve stimulation. Cortical hyperexcitability, and subsequent cortical spreading depolarisation, also occurs. This may account for the aura or visual symptoms experienced by many migraineurs (Hadjikhani 2001). There may be overlap between headache migraine pathways and those of the vestibular system, accounting for the balance symptoms. For example, the trigeminovascular system receives pain signals from nerves of the dura mater and large intracranial blood vessels, but also from vessels of the inner ear (Vass 1998). Abnormal thalamic activation in response to vestibular stimulation has also been identified in patients with vestibular migraine (Russo 2014). CGRP itself is implicated in vestibular migraine, along with headache migraine, and increased CGRP levels have been linked to the development of symptoms in migraine (Villalón 2009). Work is ongoing into the relevance of CGRP in vestibular migraine, and whether pharmacological targeting of this molecule and its receptors will affect the condition. 

The consequences of vestibular migraine for the individual may be considerable. The unpredictable, disabling attacks of spinning sensory disorientation can be distressing and debilitating in equal measure. This has a considerable impact on engagement with day‐to‐day activities and overall quality of life. 

### Description of the intervention

Current pharmacological treatments for patients with vestibular migraine may be prophylactic, or used to treat an acute attack. Many are based on interventions that have been widely used to treat headache migraine. This review is focused on non‐pharmacological interventions that are taken as prophylaxis to prevent attacks occurring. 

A number of different non‐pharmacological interventions have been proposed to be of benefit in headache migraine, and have consequently been considered for use in vestibular migraine. Many well‐recognised triggers exist for migraine attacks, and several interventions aim to reduce or eliminate these triggers. These include:

- dietary modification (including elimination of food triggers such as alcohol, caffeine or other foods);
- sleep improvement techniques (to ensure regular sleep patterns);
- vitamin and mineral supplements;
- talking therapies and stress management (such as counselling, cognitive behavioural therapy (CBT), meditation or mindfulness).

Vestibular rehabilitation may also be used for people with vestibular migraine but the mechanism by which this may work as prophylaxis is uncertain. This is an exercise‐based therapy that involves walking exercises, balance retraining, and visual and postural exercises. Exercises are tailored to the individual, to account for their specific symptoms. Vestibular rehabilitation is often provided in person, on a one‐to‐one or group basis, by a therapist. However, self‐directed booklet‐based and internet‐based packages are now available. 

### How the intervention might work

Triggers for migraine are widely recognised, but poorly understood. Although the pathophysiology of attacks is starting to be understood, the underlying mechanisms that initiate a migraine attack are still unclear. Nonetheless, it seems clear that a reduction in the presence of triggers should reduce the frequency of attacks. 

Many of the interventions included in this review are used on this basis ‐ that dietary modification, reduction in stress, regulation of sleep or nutritional status should all reduce the frequency of triggers for a migraine attack. 

Vestibular rehabilitation aims to retrain the balance system. However, it may also allow people to cope better with their vestibular symptoms during an episode. 

### Why it is important to do this review

Balance disorders can be difficult to diagnose and treat. There are few specific diagnostic tests, a variety of related disorders and a limited number of interventions that are known to be effective. To determine which topics within this area should be addressed with new or updated systematic reviews, we conducted a scoping and prioritisation process, involving stakeholders (https://ent.cochrane.org/balance-disorders-ent). Vestibular migraine was ranked as one of the highest priority topics during this process (along with persistent postural‐perceptual dizziness and Ménière's disease). 

The impact of vestibular migraine is considerable, with 40% of sufferers reporting sickness from work, and over 70% reporting the impact of their symptoms on daily activities as either moderate or severe (Neuhauser 2006). At present, there are no national or international guidelines to inform the management of this condition, therefore up‐to‐date, reliable evidence syntheses are required to help patients and healthcare professionals determine the benefits and harms of different interventions used for the condition. 

## Objectives

To assess the benefits and harms of non‐pharmacological treatments used for prophylaxis of vestibular migraine. 

## Methods

### Criteria for considering studies for this review

#### Types of studies

We included randomised controlled trials (RCTs) and quasi‐randomised trials (where trials were designed as RCTs, but the sequence generation for allocation of treatment used methods such as alternate allocation, birth dates etc). 

The number of episodes of vestibular migraine may vary with time ‐ patients sometimes have periods of more active disease, followed by a period of fewer attacks. Therefore cross‐over trials are not an appropriate study design when assessing prophylaxis for this condition. Cross‐over RCTs would only have been included if data could be extracted for the first phase of the study. If cluster‐RCTs were identified then they would have been eligible for inclusion, providing we could appropriately account for the clustering in the data analysis (according to methods described in the *Cochrane Handbook for Systematic Reviews of Interventions*) (Handbook 2021). However, we did not identify any cross‐over or cluster‐randomised trials for this review.  

#### Types of participants

We included studies that recruited participants with a diagnosis of vestibular migraine, according to the International Headache Society (IHS) and Bárány Society criteria (see Appendix 1). We also included studies that used other, established criteria, for example Neuhauser 2001 (Appendix 2). 

We included studies where participants were diagnosed with either 'definite' vestibular migraine or 'probable' vestibular migraine. 

Where studies recruited participants with a variety of diagnoses (e.g. vestibular migraine and classical migraine) we planned to include the study if either

- the majority of participants (≥ 90%) had a diagnosis of vestibular migraine; or
- subgroup data were available that allowed us to identify data specifically from those with vestibular migraine. 

However, we did not identify any trials that included participants with classical migraine. 

#### Types of interventions

We included the following interventions:

- dietary modification (including elimination of food triggers such as alcohol, caffeine or other foods, salt restriction, altered timing of meals);
- sleep improvement techniques (to ensure regular sleep patterns);
- vitamin and mineral supplements;
- herbal supplements (e.g. St John's Wort, feverfew);
- talking therapies (such as counselling or CBT);
- mind‐body interventions (e.g. meditation or mindfulness, yoga, T'ai Chi);
- vestibular rehabilitation.

We excluded interventions from alternative medical systems (e.g. Ayurvedic medicines or traditional Chinese medicines) and energy therapies (e.g. acupuncture, acupressure and magnetic therapy). We used the system developed by Wieland et al to classify complementary and alternative medicines and identify whether they will be included/excluded from the review (Wieland 2011).

The main comparisons were planned to be:

- dietary modification versus no intervention/placebo;
- sleep improvement techniques versus no intervention/placebo;
- vitamin and mineral supplements versus no intervention/placebo;
- herbal supplements versus no intervention/placebo;
- talking therapies versus no intervention/placebo;
- mind‐body interventions versus no intervention/placebo;
- vestibular rehabilitation versus no intervention/placebo.

##### Concurrent treatments

There were no limits on the type of concurrent treatments used, providing these were used equally in each arm of the study. We planned to pool studies that included concurrent treatments with those where participants did not receive concurrent treatment, and to conduct subgroup analysis to determine whether the effect estimates may be different in those receiving additional treatment. 

#### Types of outcome measures

We assessed outcomes at the following time points:

- < 3 months;
- 3 to 6 months;
- > 6 to 12 months.

The exception was for adverse event data, when we used the longest time period of follow‐up. 

We searched the COMET database for existing core outcome sets of relevance to vestibular migraine and vertigo, but were unable to find any published core outcome sets. We therefore conducted a survey of individuals with experience of (or an interest in) balance disorders to help identify outcomes that should be prioritised. The results of this survey were used by the review author team to inform the choice of outcome measures in this review.

We analysed the following outcomes in the review, but we did not use them as a basis for including or excluding studies.

##### Primary outcomes

- Improvement in vertigo
  - Measured as a dichotomous outcome (improved/not improved), according to self‐report, or according to a change of a specified score (as described by the study authors) on a vertigo rating scale.
- Change in vertigo
  - Measured as a continuous outcome, to identify the extent of change in vertigo symptoms.
- Serious adverse events
  - Including any event that caused death, was life‐threatening, required hospitalisation, resulted in disability or permanent damage, or in congenital abnormality. Measured as the number of participants who experienced at least one serious adverse event during the follow‐up period.

Vertigo symptoms comprise a variety of different features, including frequency of episodes, duration of episodes and severity/intensity of the episodes. Where possible, we included data for the vertigo outcomes that encompass all of these three aspects (frequency, duration and severity/intensity of symptoms). However, we anticipated that these data may not be available from all studies. If they were unavailable, then we extracted data on the frequency of vertigo episodes as an alternative measure for these outcomes. 

##### Secondary outcomes

- Disease‐specific health‐related quality of life
  - Measured with the Dizziness Handicap Inventory (DHI, Jacobsen 1990), a validated measurement scale in widespread use. If data from the DHI were unavailable we planned to extract data from alternative validated measurement scales, according to the order of preference described in the list below (based on the validity of the scales for this outcome):
    - DHI short form (Tesio 1999);
    - DHI screening tool (Jacobsen 1998).
  - Measured with tools to assess migraine‐related quality of life, such as the Migraine‐Specific Quality of Life Questionnaire (Jhingran 1998).
- Improvement in headache 
  - Measured as a dichotomous outcome (improved/not improved), according to self‐report, or according to a change of specified score (as described by the study authors) on a headache rating scale.
- Improvement in other migrainous symptoms 
  - Measured as a dichotomous outcome (improved/not improved), according to self‐report, or according to a change of specified score (as described by the study authors) on a rating scale.
  - Including nausea and vomiting, photophobia and phonophobia, visual aura.
- Other adverse effects
  - Including the number of participants who discontinued the intervention due to adverse effects, or for other reasons.
  - We also planned to use an exploratory approach to adverse events, and record any specific adverse events described in the studies. 

### Search methods for identification of studies

The Cochrane ENT Information Specialist conducted systematic searches for randomised controlled trials and controlled clinical trials. There were no language, publication year or publication status restrictions. The date of the search was 23 September 2022.

#### Electronic searches

The Information Specialist searched:

- the Cochrane ENT Trials Register (searched via the Cochrane Register of Studies to 23 September 2022);
- the Cochrane Central Register of Controlled Trials (CENTRAL) (searched via the Cochrane Register of Studies to 23 September 2022);
- Ovid MEDLINE(R) Epub Ahead of Print, In‐Process & Other Non‐Indexed Citations, Ovid MEDLINE(R) Daily and Ovid MEDLINE(R) (1946 to 23 September 2022);
- Ovid Embase (1974 to 23 September 2022);
- Web of Knowledge, Web of Science (1945 to 23 September 2022);
- ClinicalTrials.gov, www.clinicaltrials.gov (to 23 September 2022);
- World Health Organization (WHO) International Clinical Trials Registry Platform (ICTRP), https://trialsearch.who.int/ (to 23 September 2022).

The Information Specialist modelled subject strategies for databases on the search strategy designed for CENTRAL. The strategy has been designed to identify all relevant studies for a suite of reviews on various interventions for vestibular migraine (Webster 2022a; Webster 2022b; Webster 2022c). Where appropriate, they were combined with subject strategy adaptations of the highly sensitive search strategy designed by Cochrane for identifying randomised controlled trials and controlled clinical trials (as described in the Technical Supplement to Chapter 4 of the *Cochrane Handbook for Systematic Reviews of Interventions* version 6.1) (Lefebvre 2020). Search strategies for major databases including CENTRAL are provided in Appendix 3.

#### Searching other resources

We scanned the reference lists of identified publications for additional trials and contacted trial authors if necessary. In addition, the Information Specialist searched Ovid MEDLINE to retrieve existing systematic reviews relevant to this systematic review, so that we could scan their reference lists for additional trials. The Information Specialist also ran non‐systematic searches of Google Scholar to identify trials not published in mainstream journals.

We did not perform a separate search for adverse effects. We considered adverse effects described in included studies only.

### Data collection and analysis

#### Selection of studies

At least two review authors or co‐workers (of AD, KG, LHK, KW, SC) independently screened the remaining titles and abstracts using Covidence to identify studies that may be relevant for this review. Any discrepancies were resolved by consensus, or by retrieving the full text of the study for further assessment. 

We obtained the full text for any study that may have been relevant and two authors or co‐workers (of AD, KG, LHK, KW) again independently checked this to determine whether it met the inclusion criteria for the review. Any differences were resolved by discussion and consensus, or through recourse to a third author if necessary. 

We listed as excluded any studies that were retrieved in full text but subsequently deemed to be inappropriate for the review (according to the inclusion/exclusion criteria), according to the main reason for exclusion. 

The unit of interest for the review is the study, therefore multiple papers or reports of a single study have been grouped together under a single reference identification. We recorded the study selection process in sufficient detail to complete a PRISMA flow diagram (Figure 1) and the Characteristics of excluded studies table. 

**1:**
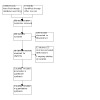
PRISMA flow chart of study retrieval and selection.

##### Screening eligible studies for trustworthiness

We assessed all studies meeting our inclusion criteria for trustworthiness using a screening tool developed by Cochrane Pregnancy and Childbirth. This tool includes specified criteria to identify studies that are considered sufficiently trustworthy to be included in the review (see Appendix 4). If any studies were assessed as being potentially 'high risk', we attempted to contact the study authors to obtain further information or address any concerns. We planned to exclude 'high risk' studies from the main analyses of the review if we were unable to contact the authors, or there was persisting uncertainty about the study, and only include studies with concerns as part of a sensitivity analysis (see Sensitivity analysis). The process is outlined in Figure 2.

**2:**
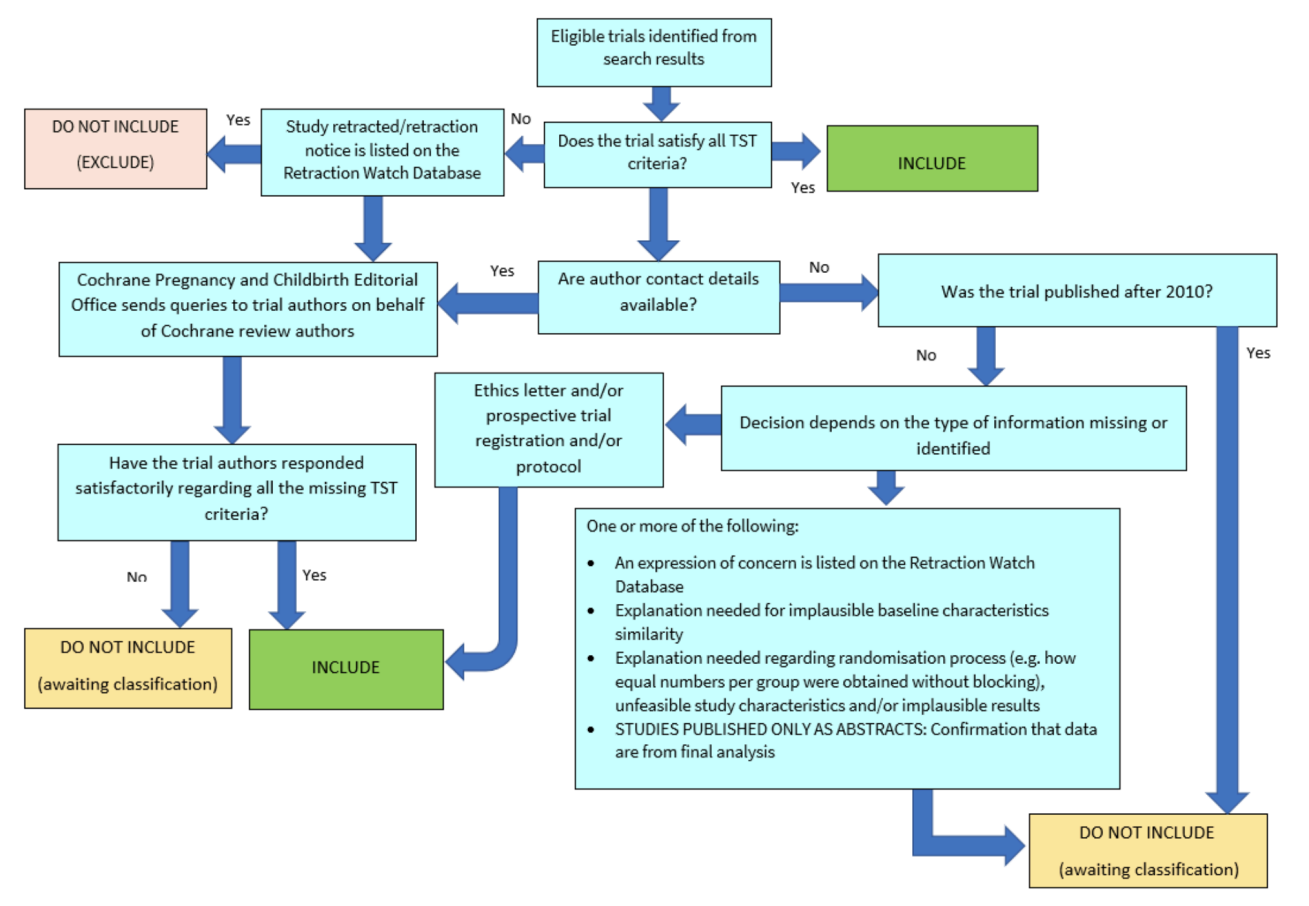
The Cochrane Pregnancy and Childbirth Trustworthiness Screening Tool

However, none of the three studies included in this review satisfied all criteria for the screening tool. We noted that the study Aydin 2020 was retrospectively registered, and that an inappropriate method was used to randomise participants to the different groups (alternate allocation). There was also a discrepancy in the trial dates reported for Qi 2020, which suggested that trial registration may have been retrospective. Finally, limited baseline data were reported by McPhee 2017, therefore we were unable to adequately compare the two groups. 

We attempted to contact authors to clarify these issues, but received no replies. We had not anticipated this issue when drafting the protocol for our review.

There are several possible explanations for the studies that had concerns when using the tool. One is that there are issues with the trustworthiness of the studies identified in this review, and the data included may not give reliable estimates of the true effect. Alternatively, the trustworthiness screening tool may be excessively sensitive, and flag studies that are trustworthy, but where information has not been fully reported. We note that this tool (and others used for the same purpose) has not yet been validated for use. 

We therefore took the decision to include the studies in the review, despite the potential concerns over trustworthiness. The uncertainty in the results is captured as part of our GRADE rating in the certainty of the evidence, using the domain 'study limitations'. 

#### Data extraction and management

At least two review authors (of AD, LHK and KW) independently extracted outcome data from each study using a standardised data collection form. Where a study had more than one publication, we retrieved all publications to ensure complete extraction of data. Any discrepancies in the data extracted by the two authors were checked against the original reports, and differences were resolved through discussion and consensus, with recourse to a third author where necessary. If required, we contacted the study authors for clarification.

We included key characteristics of the studies, including the following information:

- study design, duration of the study, number of study centres and location, study setting and dates of the study;
- information on the participants, including the number randomised, those lost to follow‐up or withdrawn, the number analysed, the age of participants, gender, features of the condition (e.g. probable or definite vestibular migraine), diagnostic criteria used, inclusion and exclusion criteria for the individual studies;
- details of the intervention, comparator and concomitant treatments or excluded medications;
- the outcomes specified and reported by the study authors, including the time points;
- funding for the study and any conflicts of interest for the study authors;
- information required to assess the risk of bias in the study and to enable GRADE assessment of the evidence.

Once the extracted data had been checked and any discrepancies resolved, a single author transferred the information to Review Manager 5 (RevMan 2020). 

The primary effect of interest for this review is the effect of treatment assignment (which reflects the outcomes of treatment for people who were assigned to the intervention) rather than a per protocol analysis (the outcomes of treatment only for those who completed the full course of treatment as planned). For the outcomes of interest in this review, we extracted findings from the studies on an available case basis, i.e. all available data from all participants at each time point, based on the treatment to which they were randomised. This was irrespective of adherence, or whether participants had received the intervention as planned.

In addition to extracting pre‐specified information about study characteristics and aspects of methodology relevant to risk of bias, we extracted the following summary statistics for each study and outcome:

- For continuous data: the mean values, standard deviation and number of patients for each treatment group at the different time points for outcome measurement. Where change‐from‐baseline data were not available, we extracted the values for endpoint data instead. If values for the individual treatment groups were not reported, where possible we extracted summary statistics (e.g. mean difference) from the studies.
- For binary data: we extracted information on the number of participants experiencing an event, and the number of participants assessed at that time point. If values for the individual treatment groups were not reported, where possible we extracted summary statistics (e.g. risk ratio) from the studies.
- For ordinal scale data: if the data appear to be normally distributed, or if the analysis performed by the investigators indicated that parametric tests are appropriate, then we treated the outcome measure as continuous data. Alternatively, if data were available, we converted these to binary data for analysis.
- For time‐to‐event data: we did not identify any time‐to‐event data for this review. 

If necessary, we converted data found in the studies to a format appropriate for meta‐analysis, according to the methods described in the *Cochrane Handbook for Systematic Reviews of Interventions* (Handbook 2021). 

We pre‐specified time points of interest for the outcomes in this review. Where studies reported data at multiple time points, we took the longest available follow‐up point within each of the specific time frames. For example, if a study reported an outcome at 16 weeks and 20 weeks of follow‐up then the 20‐week data was included for the time point three to six months (12 to 24 weeks).

#### Assessment of risk of bias in included studies

Two authors (of AD, LHK, KW) undertook assessment of the risk of bias of the included studies independently, with the following taken into consideration, as guided by the *Cochrane Handbook for Systematic Reviews of Interventions* (Handbook 2011).

- sequence generation;
- allocation concealment;
- blinding;
- incomplete outcome data;
- selective outcome reporting; and
- other sources of bias.

We used the Cochrane risk of bias tool (Handbook 2011), which involves describing each of these domains as reported in the study and then assigning a judgement about the adequacy of each entry: 'low', 'high' or 'unclear' risk of bias.

#### Measures of treatment effect

We summarised the effects of dichotomous outcomes (e.g. serious adverse effects) as risk ratios (RR) with 95% confidence intervals (CIs). We have also expressed the results as absolute numbers based on the pooled results and compared to the assumed risk in the summary of findings tables (Table 1; Table 2; Table 3) and full GRADE profiles (Table 4; Table 5; Table 6). 

**1:** GRADE profile: Dietary intervention (probiotics) versus placebo for prophylaxis of vestibular migraine

**Certainty assessment**							**№ of patients**		**Effect**		**Certainty**
**№ of studies**	**Study design**	**Risk of bias**	**Inconsistency**	**Indirectness**	**Imprecision**	**Other considerations**	**Probiotics**	**no treatment**	**Relative** **(95% CI)**	**Absolute** **(95% CI)**	
**Change in vertigo: global score (assessed with: 10‐point Likert scale. Higher scores = worse symptoms; scale from: 1 to 10)**											
1	Randomised trials	Serious^a, b^	Not serious	Serious^c^	Serious^d^	None	103	101	—	MD **0.1 points higher** (0.88 lower to 1.08 higher)	⨁◯◯◯ Very low
**Change in vertigo: global score (follow‐up: range 3 months to 6 months; assessed with: 10‐point Likert scale. Higher scores = worse symptoms; scale from: 1 to 10)**											
1	Randomised trials	Serious^a,b^	Not serious	Serious^c^	Serious^d^	None	103	101	—	MD **2.2 points lower** (3.73 lower to 0.67 lower)	⨁◯◯◯ Very low
**Change in vertigo: frequency (assessed with: number of attacks per week)**											
1	Randomised trials	Serious^a,b^	Not serious	Not serious	Serious^d^	None	103	101	—	MD **0.1 attacks per week higher** (0.61 lower to 0.81 higher)	⨁⨁◯◯ Low
**Change in vertigo: frequency (follow‐up: range 3 months to 6 months; assessed with: number of attacks per week)**											
1	Randomised trials	Serious^a,b^	Not serious	Not serious	Very serious^d,e^	None	103	101	—	MD **0.7 attacks per week lower** (2.39 lower to 0.99 higher)	⨁◯◯◯ Very low
**Change in disease‐specific health‐related quality of life (QOL) (assessed with: DHI; higher scores = worse QOL; scale from: 1 to 100)**											
1	Randomised trials	Serious^b^	Not serious	Not serious	Serious^d^	None	103	101	—	MD **5.4 points lower** (11.4 lower to 0.6 higher)	⨁⨁◯◯ Low
**Change in disease‐specific health‐related quality of life (follow‐up: range 3 months to 6 months; assessed with: DHI; scale from: 1 to 100)**											
1	Randomised trials	Serious^b^	Not serious	Not serious	Serious^d^	None	103	101	—	MD **9.6 points lower** (19.04 lower to 0.16 lower)	⨁⨁◯◯ Low

**CI:** confidence interval; **DHI:** Dizziness Handicap Inventory; **MD:** mean difference ^a^High risk of reporting bias with this outcome, as vertigo results are reported differently to the process specified in the study methods section. ^b^Concerns over lack of detail on randomisation methods, potential for variable use of additional interventions in each study group over the study period (4 months), discrepancy in trial data reporting and indication of a per protocol analysis. ^c^Unclear if measurement scale reflects vertigo itself or also includes quality of life measures. ^d^Sample size fails to meet the optimal information size (OIS) for this outcome, taken to be < 400 participants for a continuous outcome or < 300 events for a dichotomous outcome. ^e^Confidence interval includes the possibility of potential benefit, as well as possible harm from the intervention. Minimally important difference assumed to be approximately 1 attack per week.

**2:** GRADE profile: CBT versus no treatment for prophylaxis of vestibular migraine

**Certainty assessment**							**№ of patients**		**Effect**		**Certainty**
**№ of studies**	**Study design**	**Risk of bias**	**Inconsistency**	**Indirectness**	**Imprecision**	**Other considerations**	**CBT**	**No treatment**	**Relative** **(95% CI)**	**Absolute** **(95% CI)**	
**Vertigo (global score) (assessed with: VSS‐SF (higher scores = worse symptoms); scale from: 0 to 60)**											
1	Randomised trials	Very serious^a^	Not serious	Serious^b^	Very serious^c,d^	None	16	18	—	MD **1.23 points higher** (7.41 lower to 9.87 higher)	⨁◯◯◯ Very low
**Disease‐specific health‐related quality of life (related to vertigo) (assessed with: DHI (higher scores = worse symptoms); scale from: 0 to 100)**											
1	Randomised trials	Very serious^a^	Not serious	Not serious	Very serious^c^	None	16	18	—	MD **3.16 points higher** (13.27 lower to 19.59 higher)	⨁◯◯◯ Very low
**Disease‐specific health‐related quality of life (related to migraine) (assessed with: MIDAS (higher scores = worse symptoms); scale from: 0 to 270)**											
1	Randomised trials	Very serious^a^	Not serious	Not serious	Very serious^c,d^	None	16	18	—	MD **7.71 points lower** (41.15 lower to 25.73 higher)	⨁◯◯◯ Very low
**Discontinuation of allocated treatment**											
1	Randomised trials	Very serious^a^	Not serious	Not serious	Very serious^d,e^	None	16/33 (48.5%)	10/28 (35.7%)	**RR 1.36** (0.74 to 2.50)	**129 more per 1000** (from 93 fewer to 536 more)	⨁◯◯◯ Very low

**CBT:** cognitive behavioural therapy; **CI:** confidence interval; **DHI:** Dizziness Handicap Inventory; **MD:** mean difference; **MIDAS:** Migraine Disability Assessment Scale; **RR:** risk ratio; **VSS‐SF:** Vertigo Symptom Scale Short Form ^a^High risk of performance and detection bias, due to lack of blinding. High risk of attrition bias due to missing outcome data. ^b^VSS includes other symptoms (such as autonomic and anxiety symptoms) and does not specifically relate to vertigo symptoms. No data were available for the vestibular‐balance subscale. ^c^Extremely small sample size. ^d^Confidence intervals include the possibility of either benefit or harm from the intervention. ^e^Sample size fails to meet optimal information size (taken as < 300 events for a dichotomous outcome, or < 400 participants for a continuous outcome).

**3:** GRADE profile: Vestibular rehabilitation versus no treatment for prophylaxis of vestibular migraine

**Certainty assessment**							**№ of patients**		**Effect**		**Certainty**
**№ of studies**	**Study design**	**Risk of bias**	**Inconsistency**	**Indirectness**	**Imprecision**	**Other considerations**	**Vestibular rehabilitation**	**No treatment**	**Relative** **(95% CI)**	**Absolute** **(95% CI)**	
**Change in vertigo (follow‐up: range < 3 months; assessed with: median number of attacks per month)**											
1	Randomised trials	Very serious^a^	Not serious	Serious^b^	Very serious^c^	None	20	20	The median number of vertigo attacks per month was 4.5 in the VR group and 5 in the control group. No statistical analysis was presented.		⨁◯◯◯ Very low
**Change in vertigo (follow‐up: range 3 months to 6 months; assessed with: median number of attacks per month)**											
1	Randomised trials	Very serious^a^	Not serious	Serious^b^	Very serious^c^	None	20	20	The median number of vertigo attacks per month was 1.5 in the VR group and 4.5 in the control group. No statistical analysis was presented.		⨁◯◯◯ Very low
**Disease‐specific health‐related quality of life (follow‐up: range < 3 months; assessed with: DHI)**											
1	Randomised trials	Very serious^a^	Not serious	Serious^b^	Very serious^c^	None	20	20	The median DHI score was 33 in the VR group and 36 in the control group. No statistical analysis was presented		⨁◯◯◯ Very low
**Disease‐specific health‐related quality of life (follow‐up: range 3 months to 6 months; assessed with: DHI)**											
1	Randomised trials	Very serious^a^	Not serious	Serious^b^	Very serious^c^	None	20	20	The median DHI score was 20 in the VR group and 33 in the control group. No statistical analysis was presented.		⨁◯◯◯ Very low
**Change in headache (follow‐up: range < 3 months; assessed with: median number of headache attacks per month)**											
1	Randomised trials	Very serious^a^	Not serious	Serious^b^	Very serious^c^	None	20	20	The median number of headache attacks per month was 5 in the intervention group and 2.5 in the control group. The P value is reported as 0.483.		⨁◯◯◯ Very low
**Change in headache (follow‐up: range 3 months to 6 months; assessed with: median number of headache attacks per month)**											
1	Randomised trials	Very serious^a^	Not serious	Serious^b^	Very serious^c^	None	20	20	The median number of headache attacks per month was 1 in the intervention group and 0.5 in the control group. The P value is reported as 0.917.		⨁◯◯◯ Very low

**CI:** confidence interval; **DHI:** Dizziness Handicap Inventory; **VR:** vestibular rehabilitation ^a^Very serious risk of bias due to quasi‐randomised allocation, lack of blinding of study participants and outcome assessors, and attrition bias. ^b^Use of background pharmacological therapy in both groups ‐ details on the nature of this treatment are not provided. ^c^Very small study. Unable to appropriately compare groups with the data presented.

For continuous outcomes, we expressed treatment effects as a mean difference (MD) with standard deviation (SD). We did not need to present any data using a standardised mean difference in this review. 

#### Unit of analysis issues

Vestibular migraine is unlikely to be a stable condition and interventions may not have a temporary effect. If cross‐over trials were identified then we planned to use only the data from the first phase of the study. If cluster‐randomised trials were identified then we would have ensured that analysis methods were used to account for clustering in the data according to the Handbook 2021. However, neither of these study designs were identified in the included studies. 

If we had identified studies with three or more arms, we would have ensured these were included to avoid double‐counting of any participants. However, this was not necessary for this review. 

#### Dealing with missing data

We tried to contact study authors via email whenever the outcome of interest was not reported, if the methods of the study suggested that the outcome had been measured. We planned to do the same if not all data required for meta‐analysis were reported (for example, standard deviations), unless we were able to calculate them from other data reported by the study authors. 

#### Assessment of heterogeneity

We planned to assess clinical heterogeneity by examining the included studies for potential differences between them in the types of participants recruited, interventions or controls used and the outcomes measured. However, all of the included studies assessed different interventions, therefore this was not really appropriate. 

We also planned to use the I^2^ statistic to quantify inconsistency among the studies in each analysis, and to considered the P value from the Chi^2^ test. However, a single study contributed data to each of the comparisons in this review, so it was not possible to assess statistical heterogeneity in this way. 

#### Assessment of reporting biases

We assessed reporting bias as within‐study outcome reporting bias and between‐study publication bias.

##### Outcome reporting bias (within‐study reporting bias)

We assessed within‐study reporting bias by comparing the outcomes reported in the published report against the study protocol or trial registry, whenever this could be obtained. If the protocol or trial registry entry was not available, we compared the outcomes reported to those listed in the methods section. If results were mentioned but not reported adequately in a way that allows analysis (e.g. the report only mentions whether the results were statistically significant or not), bias in a meta‐analysis is likely to occur. We planned to seek further information from the study authors in this situation. If no further information could be found, we noted this as being a 'high' risk of bias when the risk of bias tool was used. If there was insufficient information to judge the risk of bias we noted this as an 'unclear' risk of bias  (Handbook 2011). 

##### Publication bias (between‐study reporting bias)

We planned to assess funnel plots if sufficient studies (more than 10) were available for an outcome. However, we did not identify sufficient studies to enable this. We did not identify any unpublished studies as part of this review. 

#### Data synthesis

##### Meta‐analysis of numerical data

The three studies included in this review all assessed different interventions, therefore we were unable to pool any data in a meta‐analysis. Instead, we present the results of individual studies. 

##### Synthesis using other methods

If we were unable to pool numerical data in a meta‐analysis for one or more outcomes we planned to provide a synthesis of the results using alternative methods, following the guidance in Chapter 12 of the Handbook 2021. However, as noted above, all of the studies assessed different interventions, therefore synthesis of the results was not required. 

#### Subgroup analysis and investigation of heterogeneity

We planned to assess statistical heterogeneity considering the following subgroups:

- Different types of intervention, within a specific group.
- Use of any concomitant treatment.
- Diagnosis of vestibular migraine.
- Age of the participants.
- Sex of the participants.

However, due to the paucity of data available, and as no meta‐analyses were included in this review, we did not carry out any subgroup analysis. 

#### Sensitivity analysis

We planned to carry out a number of sensitivity analyses for the primary outcomes in this review. However, the paucity of data and the lack of meta‐analyses has meant that this was not possible. 

We used the Cochrane Pregnancy and Childbirth Screening Tool to identify any studies with concerns over the data available. We had intended that any studies identified by the tool would be excluded from the main analyses in the review, but that we would explore the impact of including the data from these studies through a sensitivity analysis. However, as noted above, we had some concerns over the use of this tool, and few studies were included in the review, therefore this sensitivity analysis was not conducted. 

#### Summary of findings and assessment of the certainty of the evidence

Two independent authors (KG, KW) used the GRADE approach to rate the overall certainty of evidence using GRADEpro GDT (https://gradepro.org/) and the guidance in Chapter 14 of the *Cochrane Handbook for Systematic Reviews of Interventions* (Handbook 2021). Disagreements were resolved through discussion and consensus, or with recourse to a third author if necessary. The certainty of evidence reflects the extent to which we are confident that an estimate of effect is correct and we applied this in the interpretation of results. There are four possible ratings: high, moderate, low and very low. A rating of high certainty of evidence implies that we are confident in our estimate of effect and that further research is very unlikely to change our confidence in the estimate of effect. A rating of very low certainty implies that any estimate of effect obtained is very uncertain.

The GRADE approach rates evidence from RCTs that do not have serious limitations as high certainty. However, several factors can lead to the downgrading of the evidence to moderate, low or very low. The degree of downgrading is determined by the seriousness of these factors:

- Study limitations (risk of bias):
  - This was assessed using the rating from the Cochrane risk of bias tool for the study or studies included in the analysis. We rated down either one or two levels, depending on the number of domains that had been rated at high or unclear risk of bias. 
- Inconsistency:
  - This was assessed using the I^2^ statistic and the P value for heterogeneity for all meta‐analyses, as well as by visual inspection of the forest plot. For results based on a single study we rated this domain as no serious inconsistency.
- Indirectness of evidence:
  - We took into account whether there were concerns over the population included in the study or studies for each outcome, as well as whether additional treatments were offered that may impact on the efficacy of the intervention under consideration. 
- Imprecision:
  - We took into account the sample size and the width of the confidence interval for each outcome. If the sample size did not meet the optimal information size (i.e. < 400 people for continuous outcomes or < 300 events for dichotomous outcomes), or the confidence interval crossed the small effect threshold we rated down one level. If the sample size did not meet the optimal information size and the confidence interval included both potential harm and potential benefit we rated down twice. We also rated down twice for very tiny studies (e.g. 10 to 15 participants in each arm), regardless of the estimated confidence interval.
- Publication bias:
  - We considered whether there were likely to be unpublished studies that may impact on our confidence in the results obtained. 

We used a minimally contextualised approach, and rated the certainty in the interventions having an important effect (Zeng 2021). Where possible, we used agreed minimally important differences (MIDs) for continuous outcomes as the threshold for an important difference. Where no MID was identified, we provide an assumed MID based on agreement between the authors. For dichotomous outcomes, we looked at the absolute effects when rating imprecision, but also took into consideration the GRADE default approach (rating down when a RR crosses 1.25 or 0.80). We have justified all decisions to downgrade the certainty of the evidence using footnotes, and added comments to aid the interpretation of the findings, where necessary. 

In the protocol for this review we planned to provide a summary of findings table for the following comparisons (Webster 2022c):

- dietary modification versus no intervention/placebo;
- vitamin and mineral supplements versus no intervention/placebo.

However, we only found data on one dietary intervention (probiotics) and we did not find any studies of vitamin or mineral supplements. We considered that, in the absence of other data, the results of the three comparisons in this review would be of interest to consumers (both people with vestibular migraine and healthcare professionals), therefore we have reported a summary of findings table for each of these comparisons:

- probiotics versus no treatment for vestibular migraine;
- CBT versus no treatment for vestibular migraine;
- vestibular rehabilitation versus no treatment for vestibular migraine.

We included all primary outcomes in the summary of findings table and prioritised outcomes at the time point three to six months for presentation in the table. However, as some outcomes were only reported at earlier time points, these were also included. We have also included a full GRADE profile for all results and comparisons (Table 4; Table 5; Table 6).

## Results

### Description of studies

#### Results of the search

The searches in September 2022 retrieved a total of 1186 records. This reduced to 558 after the removal of duplicates. We screened the titles and abstracts of the remaining 558 records. We discarded 538 records and assessed 20 full‐text records, which were linked to 17 studies. 

We excluded 12 studies (12 records) with reasons recorded in the review (see Excluded studies and Characteristics of excluded studies). We identified two ongoing studies (two records), which are listed in Characteristics of ongoing studies. We included three completed studies (six records) where results were available.

A flow chart of study retrieval and selection is provided in Figure 1.

#### Included studies

We included three studies in this review (Aydin 2020; McPhee 2017; Qi 2020). Details of the individual studies can be found in the Characteristics of included studies.

##### Study design

All of the included studies were described as randomised controlled trials. Two studies included two arms, comparing an active intervention to a placebo (Qi 2020), or to no intervention (McPhee 2017). The third study included three arms, although only two arms were relevant for this review (Aydin 2020). The duration of treatment ranged from eight weeks (McPhee 2017) to six months (Aydin 2020). The largest study was Qi 2020, which recruited a total of 218 participants. 

##### Participants

All three studies recruited adult participants with a diagnosis of vestibular migraine. 

###### Diagnosis of vestibular migraine

The studies Qi 2020 and Aydin 2020 appeared to use the IHS and Bárány Society criteria for the diagnosis of vestibular migraine (see Appendix 1 for details). Qi 2020 only included those with 'definite' vestibular migraine. It was not clear whether individuals with 'probable' vestibular migraine were also included in Aydin 2020. 

McPhee 2017 used the criteria proposed by Neuhauser 2001 (see Appendix 2), and included those with either 'probable' or 'definite' vestibular migraine. 

###### Features of vestibular migraine

Two studies gave some information on the duration of disease. Participants in McPhee 2017 had a mean time since diagnosis of 7.99 months in the control group and 15.05 months in the intervention group. There was a discrepancy in the reporting of the duration of symptoms in Aydin 2020. Data reported in a table indicated that the time since onset of headache symptoms was 12.5 years, and the time since onset of vestibular symptoms was 4.75 years. However, the text stated "The patients reported having headaches for about 10 years, and the duration of the vertigo symptoms was approximately 2.8 years."

##### Interventions and comparisons

Each of the studies considered a different comparison of interest in this review. 

###### Dietary intervention (probiotics) versus placebo

The study Qi 2020 considered a dietary intervention (the use of probiotics) to placebo. Participants were randomised to receive either 2 × 10^10^ colony forming units of *Lactobacillus casei Shirota* daily for four months, or a corn‐starch placebo. 

###### Cognitive behavioural therapy (CBT) versus no treatment

McPhee 2017 used a CBT intervention for eight weeks and compared this to no treatment (a wait‐list control group).

###### Vestibular rehabilitation versus no treatment

The study Aydin 2020 included three groups. The first group received "standard medical treatment" ‐ this was not standardised across the group, but typically included propranolol (or other drugs if this was contraindicated). The second group received standard medical treatment plus vestibular rehabilitation. These groups have been compared in this review (i.e. the comparison is of vestibular rehabilitation plus standard medical care versus standard medical care alone ‐ overall, estimating the effect of vestibular rehabilitation). The third arm received vestibular rehabilitation alone, but there was no relevant arm to compare this (no group received no treatment), therefore we excluded this treatment arm from the review.  

##### Outcomes

###### 1. Improvement in vertigo

For this outcome we included dichotomous data, assessed as the proportion of participants whose vertigo had 'improved' or 'not improved'. 

####### 1.1. Global score

None of the included studies assessed the improvement in vertigo using a global score.

####### 1.2. Frequency

This outcome was not reported by any of the studies. We note that the methods section of Qi 2020 indicated that vertigo would be assessed as a categorical outcome: "The reduction in the attack number was categorized as complete resolution, substantial control (>50% decrease), moderate control (25‐50% decrease), and minimal control (<25% decrease) with unaltered or increased frequency". However, these data are not presented in the article. 

###### 2. Change in vertigo

This outcome included data on the change in vertigo using a continuous numerical scale. 

####### 2.1. Global score

Qi 2020 reported assessing the change in vertigo using a vertigo severity score "which reflects the seriousness of vertiginous attack that negatively affects the life quality of the patients". However, it was unclear whether the scoring system used reflects vertigo symptoms or quality of life. The scoring range appeared to be from 1 to 10 (with higher scores representing worse symptoms). 

McPhee 2017 assessed change in vertigo using the Vertigo Symptom Scale Short Form (VSS‐SF). This is a 15‐item instrument that assesses symptoms over the course of one month, with a total score of 0 to 60 (higher scores represent worse symptoms). The data from this instrument actually considers both vertigo symptoms (vestibular‐balance subscale, range 0 to 32), and also some additional associated symptoms (autonomic‐anxiety subscale, range 0 to 28). As the results of the vestibular‐balance subscale were not reported, we were only able to include the total score in the analysis.

Aydin 2020 did not assess the change in vertigo using a global score. 

####### 2.2. Frequency

Qi 2020 reported on the change in the frequency of vertigo attacks, by recording the number of vertigo attacks per week. Aydin 2020 assessed the frequency of vertigo attacks per month. 

This outcome was not assessed by McPhee 2017

###### 3. Serious adverse events

It was not clear whether any of the studies systematically assessed the occurrence of serious adverse events. 

###### 4. Disease‐specific health‐related quality of life

All three studies assessed this outcome using the Dizziness Handicap Inventory (DHI). 

###### 5. Improvement in headache

This outcome was only reported by Aydin 2020. The frequency of headache attacks during the study was assessed. 

###### 6. Improvement in other migrainous symptoms

This outcome was not assessed by any of the included studies. 

###### 7. Other adverse effects

No specific adverse events were reported in any of the studies. Two of the included studies gave some information on the number of participants who discontinued treatment (Aydin 2020; McPhee 2017). 

#### Excluded studies

After assessing the full text, we excluded 12 articles (linked to 12 studies) from this review. The main reason for exclusion for each study is listed below.

Six studies were not randomised controlled trials (ACTRN12616000683437; Balci 2022; ChiCTR1800014766; Koc 2021; NCT03979677; NCT05508139).

One article was a systematic review (Byun 2021). We checked the reference list to ensure that any relevant studies were included in this review. 

Two studies considered an intervention that did not meet the inclusion criteria for this review:

- Liu 2013 assessed an intervention that included a number of different components (massage, herbal medications, physiotherapy);
- Zhang 2012 assessed a traditional Chinese medicine.

Finally, three articles compared an intervention of interest to an active comparator that was not relevant for the review (as opposed to a placebo or to no treatment):

- CTRI/2022/01/039831 is an ongoing trial that will compare "proprioceptive neuromuscular facilitation" (a type of physiotherapy) to an active range of neck motion exercises. 
- Hu 2021 compared acupuncture to treatment with venlafaxine.
- Sun 2022 compared resistance exercise to relaxation therapy. 

### Risk of bias in included studies

See Figure 3 for the risk of bias graph (our judgements about each risk of bias item presented as percentages across all included studies) and Figure 4 for the risk of bias summary (our judgements about each risk of bias item for each included study). We judged all of the included studies at high risk of bias in at least one domain. 

**3:**
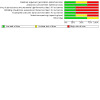
Risk of bias graph (our judgements about each risk of bias item presented as percentages across all included studies).

**4:**
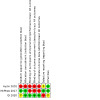
Risk of bias summary (our judgements about each risk of bias item for each included study).

#### Allocation

The method used for randomisation and allocation concealment varied across the studies. We judged McPhee 2017 at low risk of bias for this domain, as computerised randomisation was used and allocation was conducted by a third party. The methods used by Qi 2020 were not fully described, although the description of the randomisation process may indicate that computerised randomisation was used. We judged this to be at unclear risk of bias from the randomisation process, but at low risk of bias from the allocation process, which appeared to be independent. Finally, we considered the process used by Aydin 2020 to result in a high risk of bias. Participants were allocated to groups alternately, therefore the next group allocation was entirely predictable. 

#### Blinding

Only the study Qi 2020 (assessing the efficacy of probiotics) used a placebo‐controlled design. We therefore considered this study to be at low risk of performance and detection bias. The studies Aydin 2020 and McPhee 2017 used interventions for which it is difficult to blind participants (vestibular rehabilitation and cognitive behavioural therapy). The comparator groups in these studies received no intervention, therefore we considered the data to be at risk of bias for these domains. 

#### Incomplete outcome data

The studies Aydin 2020 and McPhee 2017 both had substantial dropout over the course of the study. We considered that the extent of dropout was sufficient to have a potential impact on the results, therefore we rated this domain at high risk of bias. Dropout was much more limited for the study Qi 2020, therefore we rated this study at low risk of bias. 

#### Selective reporting

We rated the study Aydin 2020 at unclear risk of bias for this domain. We identified a protocol for this study, and all outcomes appear to have been reported in accordance with this protocol. However, we also noted that the protocol appeared to have been retrospectively registered, and that statistical analyses of the data were only reported for some outcomes. We rated McPhee 2017 at low risk of bias ‐ outcomes were all reported as pre‐specified in the study protocol. We were unable to identify a published protocol for the study Qi 2020. In addition, we noted a discrepancy in the reporting of one outcome between the methods section and the results. The authors stated that vertigo would be assessed as a categorical outcome, according to the percentage reduction in the number of vertigo attacks. However, the data are instead reported on a continuous scale, comparing the average attack number in each group. We therefore considered that there was a risk of selective reporting bias with this study.    

#### Other potential sources of bias

We did not identify any other concerns with regard to the studies Aydin 2020 or McPhee 2017. We did note a number of other issues pertaining to the study Qi 2020, but were uncertain whether these would contribute a risk of bias in the overall results, therefore we have rated this domain at unclear risk of bias. Firstly, the study lasted for four months ‐ during this time participants were also offered additional medication as needed to treat their symptoms. There is no report in the article of the additional medications that were used by each group either at baseline, or for the duration of the trial. Therefore we are unable to assess whether there may have been important differences in the characteristics of trial participants at baseline, or in how they were treated during the study. We also had concerns over a discrepancy in the reported trial dates. The publication states that participants were enrolled between January 2017 and December 2018. However, the trial registry record states that prospective registration took place (on 12 June 2020) but records the date of ethical approval as 29 May 2020 and the study dates as 15 June 2020 to 10 June 2022. Finally, the authors state that a per protocol (rather than intention‐to‐treat, ITT) analysis was conducted. However, there are few data points missing from the analysis, therefore it is unclear whether a per protocol analysis actually occurred (and all participants received the intervention or placebo exactly as planned for the entire study duration), or whether an ITT analysis was actually conducted. 

### Effects of interventions

See: Table 1; Table 2; Table 3

#### Probiotics versus placebo

A single study compared the use of probiotics to placebo (Qi 2020). Follow‐up occurred at two months and four months. 

##### Improvement in vertigo

This outcome was not reported by Qi 2020, despite the methods section of the study indicating that vertigo would be assessed as a categorical outcome.

##### Change in vertigo

###### Global score

The authors report assessing the change in vertigo using a vertigo severity score "which reflects the seriousness of vertiginous attack that negatively affects the life quality of the patients". There were no details about the scoring system used and whether it reflects vertigo symptoms or quality of life. We have attempted to contact the authors to clarify this, but have not received a reply as yet. Therefore we have considered this a potential source of indirectness when using the GRADE criteria. At baseline, the median scores were 9 (out of 10) in the probiotic group and 8 in the placebo group. 

####### At < 3 months

After two months of follow‐up the mean difference in vertigo global score for those receiving probiotics was an increase of 0.10 points (95% confidence interval (CI) ‐0.88 to 1.08; 1 study; 204 participants; Analysis 1.1; very low‐certainty evidence). 

####### At 3 to 6 months

After four months of follow‐up the mean difference in vertigo global score for those receiving probiotics was a reduction of ‐2.20 points (95% CI ‐3.73 to ‐0.67; 1 study; 204 participants; Analysis 1.1; very low‐certainty evidence). It is unclear what the minimally important difference is for this unvalidated vertigo scoring system, however we anticipated that a change of at least 1 point would be meaningful to participants.

###### Frequency of vertigo

The same study reported on the frequency of vertigo episodes. At baseline, the median frequency was 2.1 attacks per week in the probiotic group and 1.9 in the placebo group.

####### At < 3 months

After two months of follow‐up the mean difference in the number of vertigo attacks per week for those receiving probiotics was 0.10 attacks per week higher than the placebo group (95% CI ‐0.61 to 0.81; 1 study; 204 participants; Analysis 1.2; low‐certainty evidence). 

####### At 3 to 6 months

After four months of follow‐up the mean difference in the number of vertigo attacks per week for those receiving probiotics was ‐0.70 episodes per week lower than those receiving placebo (95% CI ‐2.39 to 0.99; 1 study; 204 participants; Analysis 1.2; very low‐certainty evidence). It is unclear what the minimally important difference is, however we anticipated that a change of at least 1 episode per week would be meaningful to participants.

##### Serious adverse events

The authors of Qi 2020 stated that "No side or adverse effects were observed in any patients during the entire study period". However, it is unclear whether data on potential adverse events were systematically collected and recorded during the study. 

##### Disease‐specific health‐related quality of life

Qi 2020 assessed quality of life using the Dizziness Handicap Inventory (DHI), a widely used scale with a range of possible scores from 0 to 100. Higher scores indicate a worse quality of life.

###### At < 3 months

After two months of follow‐up the mean difference in DHI score for those receiving probiotics was ‐5.40 points (95% CI ‐11.40 to 0.60; 1 study; 204 participants; Analysis 1.3; low‐certainty evidence). 

###### At 3 to 6 months

After four months of follow‐up the mean difference in DHI score for those receiving probiotics was ‐9.60 points (95% CI ‐19.04 to ‐0.16; 1 study; 204 participants; Analysis 1.3; low‐certainty evidence). The minimally important difference (MID) for the DHI has been variously reported as either 11 or 18 points (Jacobsen 1990; Tamber 2009), therefore this effect size may be regarded as a trivial or small difference. 

##### Improvement in headache

This outcome was not reported by Qi 2020. 

##### Improvement in other migrainous symptoms

This outcome was not reported by Qi 2020. 

##### Other adverse effects

As described above, the authors of Qi 2020 stated that "No side or adverse effects were observed in any patients during the entire study period". However, it is unclear whether data on potential adverse events were systematically collected and recorded during the study. The number of participants who discontinued the intervention is also not reported. 

#### Cognitive behavioural therapy (CBT) versus no treatment

A single study considered this comparison (McPhee 2017). 

##### Improvement in vertigo

This outcome was not assessed. 

##### Change in vertigo

The change in vertigo was assessed using the Vertigo Symptom Scale Short Form (VSS‐SF). The data from this instrument includes both vertigo symptoms and some additional associated symptoms (autonomic‐anxiety symptoms). We have only been able to include the total score in this analysis, and we considered this as indirectness in the evidence when using the GRADE approach. 

###### Global score

####### < 3 months

The mean difference in VSS‐SF score at eight weeks was an increase (worsening) of 1.23 points for those who received CBT (95% CI ‐7.41 to 9.87 points; range 0 to 60, higher scores = worse symptoms; 1 study; 34 participants; Analysis 2.1; very low‐certainty evidence). 

###### Frequency of vertigo

This outcome was not assessed. 

##### Serious adverse events

The authors of McPhee 2017 stated that "Although the treatment intervention we tested has been shown to be acceptable and effective for those who completed it we cannot deny the possibility that individuals within the sample may have had adverse responses." It does not appear that data on adverse events were systematically collected during the study. 

##### Disease‐specific health‐related quality of life

McPhee 2017 assessed quality of life as related to vertigo symptoms (using the DHI) and migraine symptoms (using the Migraine Disability Assessment (MIDAS)). 

###### Vertigo‐related quality of life

####### < 3 months

The mean difference in DHI score at eight weeks was an increase (worsening) of 3.16 points for those receiving CBT (95% CI ‐13.27 to 19.59; 1 study; 34 participants; Analysis 2.2; very low‐certainty evidence). 

###### Migraine‐related quality of life

####### < 3 months

The mean difference in MIDAS score at eight weeks was an improvement of ‐7.71 points for those receiving CBT (95% CI ‐41.15 to 25.73; 1 study; 34 participants; Analysis 2.3; very low‐certainty evidence). This score has a range of 0 to 270, with higher scores representing worse symptoms. The minimally important difference has been suggested to be 4.5 points (Carvalho 2021). 

##### Improvement in headache

This outcome was not reported. 

##### Improvement in other migrainous symptoms

This outcome was not reported. 

##### Other adverse effects

As described above, it does not appear that data on adverse effects were systematically collected and assessed as part of this study. The authors do describe the number of participants who did not receive their allocated intervention (either CBT or wait‐list). The risk ratio for discontinuation in the CBT group compared to wait‐list control was 1.36 (95% CI 0.74 to 2.50; 1 study; 61 participants; Analysis 2.4; very low‐certainty evidence). The authors comment that this was partly because participants in the CBT group were unable to commit to the eight‐week programme. 

#### Vestibular rehabilitation versus no treatment

A single study considered this comparison (Aydin 2020). Most of the data were reported as medians and ranges, therefore we have been unable to conduct any further analysis of the data. For completeness, we present the results as reported in the study, including P values (where they were described). 

##### Improvement in vertigo

This outcome was not assessed. 

##### Change in vertigo

Change in vertigo was assessed using the frequency of attacks only. 

###### Global score

This outcome was not reported.

###### Frequency of vertigo

####### < 3 months

The median frequency of vertigo attacks in the vestibular rehabilitation group was 4.5 per month, with a range of 0 to 10, as compared to a median of 5 and a range of 0 to 30 in the placebo group (mean difference and confidence interval not estimable; 1 study; 40 participants; Analysis 3.1; very low‐certainty evidence). No statistical comparison of these data was reported. 

####### 3 to 6 months

The median frequency of vertigo attacks in the vestibular rehabilitation group was 1.5 per month, with a range of 0 to 10, as compared to a median of 4.5 and a range of 0 to 30 in the placebo group (mean difference and confidence interval not estimable; 1 study; 40 participants; Analysis 3.1; very low‐certainty evidence). No statistical comparison of these data was reported. 

##### Serious adverse events

The authors of Aydin 2020 stated that "No adverse events were observed". However, it is unclear whether data on potential adverse events were systematically collected and recorded during the study. 

##### Disease‐specific health‐related quality of life

This was assessed with the DHI, but was also only reported using medians and full ranges for the data. 

###### < 3 months

The median DHI score in the vestibular rehabilitation group was 33, with a range of 16 to 80, as compared to a median of 36 and a range of 10 to 70 in the placebo group (mean difference and confidence interval not estimable; 1 study; 40 participants; Analysis 3.2; very low‐certainty evidence). No statistical comparison of these data was reported. 

###### 3 to 6 months

The median DHI score in the vestibular rehabilitation group was 20, with a range of 0 to 86, as compared to a median of 33 and a range of 8 to 64 in the placebo group (mean difference and confidence interval not estimable; 1 study; 40 participants; Analysis 3.2; very low‐certainty evidence). No statistical comparison of these data was reported. 

##### Improvement in headache

Only the frequency of headache attacks was reported. This was also only reported using medians and full ranges for the data. 

###### < 3 months

The frequency of headache in the vestibular rehabilitation group was a median of 5 per month, with a range of 0 to 10, as compared to a median of 2.5 per month and a range of 0 to 10 in the placebo group. This comparison was reported with a P value of 0.483 (mean difference and confidence interval not estimable; 1 study; 40 participants; Analysis 3.3; very low‐certainty evidence). 

###### 3 to 6 months

The frequency of headache in the vestibular rehabilitation group was a median of 1 per month, with a range of 0 to 5, as compared to a median of 0.5 per month and a range of 0 to 6 in the placebo group. This comparison was reported with a P value of 0.917 (mean difference and confidence interval not estimable; 1 study; 40 participants; Analysis 3.3; very low‐certainty evidence). 

##### Improvement in other migrainous symptoms

This outcome was not reported. 

##### Other adverse effects

As described above, the authors of Aydin 2020 stated that "No adverse events were observed". However, it is unclear whether data on potential adverse events were systematically collected and recorded during the study. No information was provided on those who discontinued the intervention. The authors state that 17 participants dropped out of the study, but it is unclear to which group these participants had been allocated. 

## Discussion

### Summary of main results

We identified three studies for inclusion in this review, each of which addressed a different comparison. 

#### Dietary interventions (probiotics) versus placebo

One study addressed this comparison. Participants were randomised to receive either a probiotic supplement (*Lactobacillus casei Shirota*) or a corn‐starch placebo for four months. The evidence was very uncertain about the effect of probiotics on global scores of vertigo at both two months and four months follow‐up. At two months follow‐up, probiotics may make little or no difference to the number of vertigo attacks that people experience. The difference at four months was also trivial, but the evidence was very uncertain. Finally, probiotics may make little or no difference to disease‐specific health‐related quality of life, when assessed at either two or four months of follow‐up. A number of outcomes were not assessed by this study, including serious adverse events and other adverse effects, improvement in vertigo, improvement in headache and improvement in other migrainous symptoms. 

#### Cognitive behavioural therapy (CBT) versus no treatment

One study considered this comparison. CBT may make little or no difference to the change in vertigo (using the Vertigo Symptom Scale Short Form (VSS‐SF)) at eight weeks, but the evidence was very uncertain. The evidence on disease‐specific health‐related quality of life was also very uncertain ‐ this study identified a trivial difference between the groups in dizziness symptoms, and a small benefit from CBT when considering migraine (headache) symptoms at eight weeks of follow‐up. No other outcomes of interest in this review were assessed, including serious adverse events, other adverse effects, improvement in vertigo, improvement in headache and improvement in other migrainous symptoms. 

#### Vestibular rehabilitation versus no treatment

A single study considered this comparison. The article did not include a statistical comparison of the two groups for many outcomes, and insufficient data were provided for us to conduct any additional analysis. Therefore, the evidence from this study is all very uncertain. We were unable to determine if there was a difference in the frequency of vertigo, disease‐specific health‐related quality of life of frequency of headaches for those receiving vestibular rehabilitation. Other outcomes were not assessed by this study (including serious adverse events, other adverse effects, improvement in vertigo and improvement in other migrainous symptoms). 

### Overall completeness and applicability of evidence

We identified only three studies to include in this review, which addressed three different comparisons. However, there was no evidence from randomised controlled trials regarding the efficacy and harms of other comparisons of interest in this review (including other dietary modifications, changes in sleep, vitamin or mineral supplements, herbal supplements, other talking therapies or mind‐body interventions compared to no treatment or a placebo). 

We considered that all the studies included an appropriate population ‐ adult participants with vestibular migraine, as diagnosed using the criteria proposed by either the International Headache Society (IHS) and Bárány Society, or by Neuhauser 2001. However, follow‐up was conducted at between eight weeks and six months. Therefore, we do not have any information on the potential efficacy or harms of these interventions in the longer term. 

There was a complete lack of data on potential harms from these interventions. None of the studies included in this review appeared to systematically assess and report any occurrence of adverse effects. We therefore do not have any evidence regarding the possibility of harms that may be caused by these treatments. 

### Quality of the evidence

We used the GRADE approach to assess the certainty of the evidence included in this review. However, we considered all the evidence we identified to be either low‐ or very low‐certainty, meaning that we have little confidence in the estimated effects. 

We had concerns over the potential for bias in the results, due to issues in the conduct and analysis of the included studies. We acknowledge that it may be difficult to avoid a high risk of performance and detection bias when including interventions that are difficult to blind (such as CBT and vestibular rehabilitation), and outcomes that require self‐report by the participants themselves (such as vertigo and headache symptoms). Nonetheless, we also identified other issues with the included studies, which may have been avoided. This included problems with randomisation (Aydin 2020), attrition bias (McPhee 2017) and the potential for selective outcome reporting (Qi 2020). 

We had some concerns over indirectness for certain outcomes. For example, it was not clear whether the measurement scales used to assess vertigo symptoms in McPhee 2017 and Qi 2020 really considered vertigo itself, or were also intended to capture quality of life measures. Clearly agreement is needed on which symptoms are important to people with vestibular migraine, in order to establish which measurement tools should be used for future studies (and which outcomes should be prioritised in future systematic reviews).  

Imprecision in the effect estimates was also a key factor when rating the studies using GRADE. Two of the included studies were very small (Aydin 2020; McPhee 2017), and one study did not provide sufficient detail to allow comparison of the two groups (Aydin 2020). The confidence intervals for the effect estimates were either very wide, or could not be estimated, leading to imprecision in the results. 

### Potential biases in the review process

Two randomised controlled trials (RCTs) were excluded from this review as the comparator was incorrect ‐ an intervention was not compared to placebo or no treatment, but was instead compared to another (potentially) active intervention. This included one study that compared acupuncture to venlafaxine (Hu 2021), and one that compared resistance exercise to relaxation therapy (Sun 2022). The exclusion of these studies may be regarded as a source of bias in the review, although it is in accordance with our protocol (Webster 2022c). As the efficacy for different interventions in vestibular migraine is unknown, and there is no 'gold standard' treatment, we strongly felt that interventions must be compared to no treatment (or placebo) in order to accurately estimate their effects. However, future reviews may consider addressing this problem with the use of network meta‐analysis. 

As noted in Selection of studies, we intended to use the Trustworthiness Screening Tool to select studies that would be included in the main analyses in this review. However, due to the paucity of data, and some concerns over the sensitivity of the tool, we decided to include all three studies in the main analyses of this review. Nonetheless, the evidence from these studies is already rated as very low‐certainty, therefore the conclusions of this review are unlikely to be different, even if these studies were known to have problems in their conduct or reporting. 

### Agreements and disagreements with other studies or reviews

This is the first Cochrane Review on this topic. In the course of preparing this review we identified one other systematic review that evaluated the use of non‐pharmacological (and pharmacological) interventions for both the prophylaxis and acute treatment of vestibular migraine (Smyth 2022). The authors of this review included both randomised and non‐randomised studies, therefore the results are not directly comparable with our own review. However, their conclusions are similar, that the overall evidence base for the treatment of vestibular migraine is of low certainty and that well‐designed clinical trials are required in this area. 

## Authors' conclusions

**Implications for practice**

There is sparse evidence regarding non‐pharmacological interventions for the prophylaxis of vestibular migraine. Many of our comparisons of interest in this review have not been assessed in any placebo‐controlled randomised trials. We included three studies in this review, which each assessed different comparison: a dietary intervention (probiotics), cognitive behavioural therapy and vestibular rehabilitation versus placebo or no intervention. The evidence for each of them was of low or very low certainty. Therefore, we have little confidence in the results and cannot be sure whether any of these interventions are effective for the prophylaxis of vestibular migraine. None of the included studies reported on possible adverse effects, therefore we have no information on the potential for harm from these interventions. The evidence base for decision‐makers is therefore extremely limited. 

**Implications for research**

This review was conducted as part of a suite, which evaluate different interventions for the prophylaxis or acute treatment of vestibular migraine (Webster 2022a; Webster 2022b; Webster 2022c). The conclusions below relate to evidence from across the entire suite:

- There is a paucity of randomised controlled trials in this field, where active interventions are compared to no treatment or a placebo. Given the subjective nature of symptoms of vestibular migraine, the fluctuating severity of the condition and the lack of a 'gold standard' treatment, we consider that comparison with a placebo arm is vital to allow conclusions to be drawn on the efficacy and harms of different interventions. 
- Wherever possible, trialists should ensure that participants, study personnel and outcome assessors are appropriately blinded to the intervention, to reduce the risk of performance and detection bias affecting the results of studies. For non‐pharmacological interventions, careful thought should be given to the development of an appropriate 'placebo' for the control group (Furukawa 2014; Mohr 2009).
- Small, underpowered studies do little to improve the evidence base for these interventions. We would advocate the conduct of large, adequately powered, multicentre trials to ensure that more robust conclusions can be drawn from the study results. In addition, trialists need to be aware that there is considerable attrition over the course of these studies, and should be prepared to make additional efforts to improve follow‐up. 
- Future studies should also aim to follow up participants for longer periods of time, to identify whether interventions have lasting effects.
- There needs to be consensus on the appropriate outcomes to measure in trials that evaluate interventions for vestibular migraine, with input from different stakeholders, especially including those with the condition. As well as agreeing the types of outcomes that are important, the methods with which these are measured should be considered, including the use of validated scales to assess more subjective outcomes. This would be best achieved with the development of a core outcome set, analogous to that developed for use in trials of headache migraine (Haywood 2021). 

## History

Protocol first published: Issue 3, 2022

## Appendices

### Appendix 1. International Headache Society (IHS) and Bárány Society criteria for the diagnosis of vestibular migraine

From Lempert 2012:

**Vestibular migraine**

A. At least five episodes with vestibular symptoms of moderate or severe intensity, lasting five minutes to 72 hours.

B. Current or previous history of migraine with or without aura according to the International Classification of Headache Disorders (ICHD).

C. One or more migraine features with at least 50% of the vestibular episodes:

- headache with at least two of the following characteristics: one sided location, pulsating quality, moderate or severe pain intensity, aggravation by routine physical activity;
- photophobia and phonophobia;
- visual aura.

D. Not better accounted for by another vestibular or ICHD diagnosis.

**Probable vestibular migraine**

A. At least five episodes with vestibular symptoms of moderate or severe intensity, lasting five minutes to 72 hours.

B. Only one of the criteria B and C for vestibular migraine is fulfilled (migraine history *or *migraine features during the episode).

C. Not better accounted for by another vestibular or ICHD diagnosis.

To note: relevant vestibular symptoms are given as spontaneous vertigo, positional vertigo, visually induced vertigo, head motion‐induced vertigo or head motion‐induced dizziness with nausea. Moderate or severe symptoms are those which interfere with, and may prohibit, daily activities. 

### Appendix 2. Neuhauser criteria for migrainous vertigo

**Definite migrainous vertigo**

- Episodic vestibular symptoms of at least moderate severity (rotational vertigo, other illusory self or object motion, positional vertigo, head motion intolerance, i.e., sensation of imbalance or illusory self or object motion that is provoked by head motion) 
- Migraine according to the IHS criteria
- At least one of the following migrainous symptoms during at least two vertiginous attacks: 
  - migrainous headache; 
  - photophobia;
  - phonophobia; 
  - visual or other auras 
- Other causes ruled out by appropriate investigations

**Probable migrainous vertigo**

- Episodic vestibular symptoms of at least moderate severity (rotational vertigo, other illusory self or object motion, positional vertigo, head motion intolerance)
- At least one of the following: 
  - migraine according to the criteria of the IHS; 
  - migrainous symptoms during vertigo; 
  - migraine‐specific precipitants of vertigo, e.g., specific foods, sleep irregularities, hormonal changes; 
  - response to antimigraine drugs
- Other causes ruled out by appropriate investigations

Taken from Neuhauser 2001.

### Appendix 3. Search strategies

The search strategies were designed to identify all relevant studies for a suite of reviews on various interventions for vestibular migraine.

**Table:**

**CENTRAL (CRS)**	**Cochrane ENT Register (CRS)**	**MEDLINE (Ovid)**
1 MeSH DESCRIPTOR Migraine Disorders Explode All AND CENTRAL:TARGET 2 MeSH DESCRIPTOR Vestibular Diseases AND CENTRAL:TARGET 3 MeSH DESCRIPTOR Vertigo AND CENTRAL:TARGET 4 MeSH DESCRIPTOR Dizziness Explode All AND CENTRAL:TARGET 5 #2 OR #3 OR #4 AND CENTRAL:TARGET 6 #1 AND #5 AND CENTRAL:TARGET 7 (migrain* adj5 (vertig* or dizz* or vestibul* or spinning)):AB,EH,KW,KY,MC,MH,TI,TO AND CENTRAL:TARGET 8 #7 OR #6 AND CENTRAL:TARGET	1 MeSH DESCRIPTOR Migraine Disorders Explode All AND INREGISTER 2 MeSH DESCRIPTOR Vestibular Diseases AND INREGISTER 3 MeSH DESCRIPTOR Vertigo AND INREGISTER 4 MeSH DESCRIPTOR Dizziness Explode All AND INREGISTER 5 #2 OR #3 OR #4 AND INREGISTER 6 #1 AND #5 AND INREGISTER 7 (migrain* adj5 (vertig* or dizz* or vestibul* or spinning)):AB,EH,KW,KY,MC,MH,TI,TO AND INREGISTER 8 #7 OR #6 AND INREGISTER 9 * AND CENTRAL:TARGET 10 #8 NOT #9	1 exp Migraine Disorders/ 2 Vestibular Diseases/ 3 Vertigo/ 4 exp Dizziness/ 5 2 or 3 or 4 6 1 and 5 7 (migrain* adj5 (vertig* or dizz* or vestibul* or spinning)).ab,ti. 8 6 or 7 9 randomized controlled trial.pt. 10 controlled clinical trial.pt. 11 randomized.ab. 12 placebo.ab. 13 drug therapy.fs. 14 randomly.ab. 15 trial.ab. 16 groups.ab. 17 9 or 10 or 11 or 12 or 13 or 14 or 15 or 16 18 exp animals/ not humans.sh. 19 17 not 18 20 8 and 19
**Embase (Ovid)**	**Web of Science Core Collection (Web of Knowledge)**	**Trial Registries**
1. exp vestibular migraine/ 2. (migrain* adj5 (vertig* or dizz* or vestibul* or spinning)).ab,ti. 3. 1 or 2 4. Randomized controlled trial/ 5. Controlled clinical study/ 6. Random$.ti,ab. 7. randomization/ 8. intermethod comparison/ 9. placebo.ti,ab. 10. (compare or compared or comparison).ti. 11. ((evaluated or evaluate or evaluating or assessed or assess) and (compare or compared or comparing or comparison)).ab. 12. (open adj label).ti,ab. 13. ((double or single or doubly or singly) adj (blind or blinded or blindly)).ti,ab. 14. double blind procedure/ 15. parallel group$1.ti,ab. 16. (crossover or cross over).ti,ab. 17. ((assign$ or match or matched or allocation) adj5 (alternate or group$1 or intervention$1 or patient$1 or subject$1 or participant$1)).ti,ab. 18. (assigned or allocated).ti,ab. 19. (controlled adj7 (study or design or trial)).ti,ab. 20. (volunteer or volunteers).ti,ab. 21. human experiment/ 22. trial.ti. 23. 4 or 5 or 6 or 7 or 8 or 9 or 10 or 11 or 12 or 13 or 14 or 15 or 16 or 17 or 18 or 19 or 20 or 21 or 22 24. (random$ adj sampl$ adj7 ("cross section$" or questionnaire$1 or survey$ or database$1)).ti,ab. 25. comparative study/ or controlled study/ 26. randomi?ed controlled.ti,ab. 27. randomly assigned.ti,ab. 28. 25 or 26 or 27 29. 24 not 28 30. Cross‐sectional study/ 31. randomized controlled trial/ or controlled clinical study/ or controlled study/ 32. (randomi?ed controlled or control group$1).ti,ab. 33. 31 or 32 34. 30 not 33 35. (((case adj control$) and random$) not randomi?ed controlled).ti,ab. 36. (Systematic review not (trial or study)).ti. 37. (nonrandom$ not random$).ti,ab. 38. "Random field$".ti,ab. 39. (random cluster adj3 sampl$).ti,ab. 40. (review.ab. and review.pt.) not trial.ti. 41. "we searched".ab. 42. review.ti. or review.pt. 43. 41 and 42 44. "update review".ab. 45. (databases adj4 searched).ab. 46. (rat or rats or mouse or mice or swine or porcine or murine or sheep or lambs or pigs or piglets or rabbit or rabbits or cat or cats or dog or dogs or cattle or bovine or monkey or monkeys or trout or marmoset$1).ti. and animal experiment/ 47. 29 or 34 or 35 or 36 or 37 or 38 or 39 or 40 or 43 or 44 or 45 48. 23 not 47 49. 3 and 48	# 3 #2 AND #1 Indexes=SCI‐EXPANDED, CPCI‐S Timespan=All years # 2 TOPIC: (((randomised OR randomized OR randomisation OR randomisation OR placebo* OR (random* AND (allocat* OR assign*) ) OR (blind* AND (single OR double OR treble OR triple) )))) Indexes=SCI‐EXPANDED, CPCI‐S Timespan=All years # 1 TOPIC: (migrain* NEAR/5 (vertig* or dizz* or vestibul* or spinning) ) Indexes=SCI‐EXPANDED, CPCI‐S Timespan=All years	**Clinicaltrials.gov** ( migraine OR migrainous ) AND ( vertigo OR dizziness OR dizzy OR vertiginous OR vestibular OR spinning ) **ICTRP** migrain* AND (vertig* OR dizz* OR vestibul* OR spinning)

### Appendix 4. Trustworthiness Screening Tool

This screening tool has been developed by Cochrane Pregnancy and Childbirth. It includes a set of predefined criteria to select studies that, based on available information, are deemed to be sufficiently trustworthy to be included in the analysis. These criteria are:

**Research governance**

- Are there any retraction notices or expressions of concern listed on the Retraction Watch Database relating to this study?
- Was the study prospectively registered (for those studies published after 2010)? If not, was there a plausible reason?
- When requested, did the trial authors provide/share the protocol and/or ethics approval letter?
- Did the trial authors engage in communication with the Cochrane Review authors within the agreed timelines?
- Did the trial authors provide IPD data upon request? If not, was there a plausible reason?

**Baseline characteristics**

- Is the study free from characteristics of the study participants that appear too similar (e.g. distribution of the mean (SD) excessively narrow or excessively wide, as noted by Carlisle 2017)?

**Feasibility**

- Is the study free from characteristics that could be implausible? (e.g. large numbers of women with a rare condition (such as severe cholestasis in pregnancy) recruited within 12 months);
- In cases with (close to) zero losses to follow‐up, is there a plausible explanation?

**Results**

- Is the study free from results that could be implausible? (e.g. massive risk reduction for main outcomes with small sample size)?
- Do the numbers randomised to each group suggest that adequate randomisation methods were used (e.g. is the study free from issues such as unexpectedly even numbers of women ‘randomised’ including a mismatch between the numbers and the methods, if the authors say ‘no blocking was used’ but still end up with equal numbers, or if the authors say they used ‘blocks of 4’ but the final numbers differ by 6)?

Studies assessed as being potentially ‘high risk’ will be not be included in the review. Where a study is classified as ‘high risk’ for one or more of the above criteria we will attempt to contact the study authors to address any possible lack of information/concerns. If adequate information remains unavailable, the study will remain in ‘awaiting classification’ and the reasons and communications with the author (or lack of) described in detail.

The process is described in full in Figure 2. 

## Data and analyses

**Comparison 1:** Dietary intervention (probiotics) versus placebo

Outcome or subgroup title	No. of studies	No. of participants	Statistical method	Effect size
1.1 Change in vertigo: global score	1		Mean Difference (IV, Random, 95% CI)	Subtotals only
1.1.1 < 3 months	1	204	Mean Difference (IV, Random, 95% CI)	0.10 [‐0.88, 1.08]
1.1.2 3 to 6 months	1	204	Mean Difference (IV, Random, 95% CI)	‐2.20 [‐3.73, ‐0.67]
1.2 Change in vertigo: frequency	1		Mean Difference (IV, Random, 95% CI)	Subtotals only
1.2.1 < 3 months	1	204	Mean Difference (IV, Random, 95% CI)	0.10 [‐0.61, 0.81]
1.2.2 3 to 6 months	1	204	Mean Difference (IV, Random, 95% CI)	‐0.70 [‐2.39, 0.99]
1.3 Disease‐specific health‐related quality of life	1		Mean Difference (IV, Random, 95% CI)	Subtotals only
1.3.1 < 3 months	1	204	Mean Difference (IV, Random, 95% CI)	‐5.40 [‐11.40, 0.60]
1.3.2 3 to 6 months	1	204	Mean Difference (IV, Random, 95% CI)	‐9.60 [‐19.04, ‐0.16]

**Comparison 2:** Cognitive behavioural therapy (CBT) versus no treatment

Outcome or subgroup title	No. of studies	No. of participants	Statistical method	Effect size
2.1 Change in vertigo: global score	1	34	Mean Difference (IV, Random, 95% CI)	1.23 [‐7.41, 9.87]
2.2 Disease‐specific health‐related quality of life: vertigo	1	34	Mean Difference (IV, Random, 95% CI)	3.16 [‐13.27, 19.59]
2.3 Disease‐specific health‐related quality of life: migraine	1	34	Mean Difference (IV, Random, 95% CI)	‐7.71 [‐41.15, 25.73]
2.4 Discontinuation of allocated treatment	1	61	Risk Ratio (M‐H, Random, 95% CI)	1.36 [0.74, 2.50]

**Comparison 3:** Vestibular rehabilitation versus no treatment

Outcome or subgroup title	No. of studies	No. of participants	Statistical method	Effect size
3.1 Change in vertigo	1		Other data	No numeric data
3.1.1 < 3 months	1		Other data	No numeric data
3.1.2 3 to 6 months	1		Other data	No numeric data
3.2 Disease‐specific health‐related quality of life	1		Other data	No numeric data
3.2.1 < 3 months	1		Other data	No numeric data
3.2.2 3 to 6 months	1		Other data	No numeric data
3.3 Change in headache	1		Other data	No numeric data
3.3.1 < 3 months	1		Other data	No numeric data
3.3.2 3 to 6 months	1		Other data	No numeric data

## Characteristics of studies

### Characteristics of included studies [ordered by study ID]

**Table:** Aydin 2020

***Study characteristics***		
Methods	Quasi‐randomised, double‐blind, parallel‐group, 3‐arm trial with 6 months duration of intervention and follow‐up (intensive vestibular rehabilitation was offered for 2 months, but participants were then encouraged to continue with exercises for the remainder of the trial) The comparison of vestibular rehabilitation plus pharmacological therapy versus pharmacological therapy alone is relevant for this review	
Participants	**Setting: ** Recruited from outpatient neurology, physical medicine, rehabilitation and otorhinolaryngology clinics from a single centre in Turkey. Study dates: January 2015 to April 2016. **Sample size:** **Number randomised:** 77 participants **Number completed:** 60 participants **Participant baseline characteristics** Baseline characteristics were only reported for the entire cohort, which includes participants randomised to vestibular rehabilitation plus pharmacological treatment, vestibular rehabilitation alone and pharmacological treatment alone (n = 60) **Age:** Mean age 43.6 years (SD not reported) **Gender:** 54 females (90%): 6 males (10%) **Probable/definite vestibular migraine: ** All participants had definite disease **Attack frequency at baseline: ** Not reported **Duration of disease:** Unclear: there is a discrepancy in the reporting of this in the article Table 1 states: "Time since onset of headache symptoms: 12.5 years. Time since onset of vestibular symptoms: 4.75 years." Article text states: "The patients reported having headaches for about 10 years, and the duration of the vertigo symptoms was approximately 2.8 years." **Inclusion criteria:** Having a diagnosis of definite vestibular migraine according to the ICHD 3rd version and accepting involvement in the study **Exclusion criteria:** History of psychiatric disorder, which might interfere with adherence to the study protocol. Other diagnosis that might possibly cause vestibular disorders (e.g. cerebellar disorders, Ménière's disease etc). Anatomical defects of the inner ear or vestibular system. Patients who are unable to stand up straight or walk. **Diagnosis of vestibular migraine:** Definite vestibular migraine, according to the ICHD version 3	
Interventions	**Intervention (number randomised not reported, n = 20 completed)** Vestibular rehabilitation (plus pharmacological therapy). An individualised exercise and rehabilitation programme was used involving the following: Adaptation exercises: participants moved their heads in a yaw rotation whilst focusing on a stationary target, then a moving target to increase gaze stability. The exercises were performed in horizontal and vertical planes, for a period of 1 minute each, 3 times a day. Substitution and balance exercises: participants followed 2 different, non‐moving objects with eye movements and were asked to rapidly alternate their focus between them. Participants worked to restore their standing balance by standing with their eyes open or closed on a progressively narrowing base of support. Standing dynamic balance exercises: participants stood or moved without walking, such as marching in place, stepping forward or backward, stepping to the side etc. Habituation exercises: these exercises involved movements and positions sufficient to cause mild‐to‐moderate symptoms during the patient's daily activities Ambulation exercises: exercises included walking forwards, backwards, sideways, along a line and around cones, depending on their functional level The exercise programme consisted of 1 session per week for a period of 8 weeks. Each session lasted approximately 30 to 45 minutes and was conducted in the rehabilitation unit. In addition to the exercises performed at the hospital, all participants were given instructions and diagrams using software that could design customised exercises for performing a home exercise programme. Home exercises were designed to take approximately 15 to 20 minutes, and performed twice a day. All participants were instructed to adhere to their therapies after the first 2 months, and their designated interventions were kept the same throughout the study. **Comparator (number randomised not reported, n = 20 completed)** No intervention (pharmacological treatment only, as described below) **Background interventions administered to all participants** Participants were assessed by a neurologist and appropriate drug options were applied based on the patients' needs and features. Propranolol was the predominant drug used, with alternatives being selected in case of contraindication.	
Outcomes	**Primary outcomes relevant to this review:** **Improvement in vertigo** Not reported **Change in vertigo** Assessed as the frequency of vertigo attacks per month at 2‐month and 6‐month follow‐up **Serious adverse events** Authors state "no adverse events were observed" but no further information is given on how these were assessed **Secondary outcomes relevant to this review:** **Disease‐specific health‐related quality of life** Assessed using the Dizziness Handicap Inventory at 2‐month and 6‐month follow‐up **Improvement in headache** No dichotomous data on improvement in headache was reported Headache attack frequency was reported at 2‐month and 6‐month follow‐up **Improvement in other migrainous symptoms ** Not reported **Other adverse effects** Authors state "no adverse events were observed" but no further information is given on how these were assessed **Other outcomes reported in the study:** Activities Specific Balance Confidence Scale Posturography	
Notes	**Research integrity checklist:** No retractions or expressions of concern were identified The trial was registered with ClinicalTrials.gov (NCT03417596) but we note that this registration was retrospective, according to the study dates Limited baseline details were reported for the participants, but no concerns were identified Some dropout is reported No implausible results were noted The numbers allocated to each group were not reported, but we note that quasi‐randomised allocation was used, based on the day of admission	
***Risk of bias***		
**Bias**	**Authors' judgement**	**Support for judgement**
Random sequence generation (selection bias)	High risk	Quote: "Patients were randomized into three groups, in the order of their admission (e.g., 1st to Group 1, 2nd to Group 2, repetitively". Comment: sequence generated by order of recruitment into trial.
Allocation concealment (selection bias)	High risk	Quote: "Patients were randomized into three groups, in the order of their admission (e.g., 1st to Group 1, 2nd to Group 2, repetitively". Comment: group allocation would have been entirely predictable due to quasi‐randomised allocation method.
Blinding of participants and personnel (performance bias) All outcomes	High risk	Comment: although the study is reported in the trial registry as "triple blinded", no description of any blinding is given. We considered that it would not be possible to blind participants to whether they were receiving vestibular rehabilitation or not, and there is no description of the use of placebo for those who were not receiving pharmacological therapy. Therefore it is likely that this is an unblinded trial.
Blinding of outcome assessment (detection bias) All outcomes	High risk	Comment: as above, we considered that it was unlikely that participants were blinded to their allocated intervention. As most outcomes were reported by the participants themselves, this gives a the potential for bias.
Incomplete outcome data (attrition bias) All outcomes	High risk	Dropout was 17/77 participants overall and it is not clear to which groups these participants were allocated.
Selective reporting (reporting bias)	Unclear risk	The study protocol is available and all of the study’s specified (primary and secondary) outcomes that are of interest in the review have been reported. However, we note that the protocol was registered retrospectively. It appears that formal comparison of the 2 groups was only reported if a 'statistically significant' result was identified. P values are only reported when they are < 0.05, and many outcomes of interest are not formally compared in the article ‐ there is only a narrative description of the results.
Other bias	Low risk	Comment: no other concerns noted.

**Table:** McPhee 2017

***Study characteristics***		
Methods	Single‐centre, parallel‐group, open‐label RCT with 8 weeks duration of treatment and follow‐up. This trial also offered a further 3‐month extension phase, where participants in the wait‐list control group crossed over to receive treatment. Data from this period are not included in the review as they do not preserve the randomisation for the trial.	
Participants	**Setting: ** Recruitment was from a single centre (the Balance Clinic at the Royal Prince Alfred Hospital (RPAH), Camperdown) in Sydney, Australia **Sample size:** **Number randomised: ** 61 participants **Number completed: ** 34 participants **Participant baseline characteristics** **Age: ** CBT group: 41.73 years (SD 11.33) Wait‐list control: 51.32 years (SD 13.32) **Gender: ** Only reported for the entire cohort, not for each group: 44/61 female (72%) 17/61 male (28%) **Probable/definite vestibular migraine: ** Either probable or definite migraine; the number with each diagnosis was not reported **Attack frequency at baseline: ** Not reported **Duration of disease: ** CBT group: mean time since diagnosis: 15.05 months (SD 23.42) Wait‐list control: mean time since diagnosis 7.99 months (SD 11.44) **Inclusion criteria:** People who have been diagnosed by a consultant neurologist with "definite" or "probable" vestibular migraine based on the criteria of Neuhauser 2001. Aged 18 to 75 years. **Exclusion criteria:** People with a history of complex mental illness or a current substance abuse problem. People with a current diagnosis of another neurological disorder were also excluded, or considered for the vestibular control group (in a separate study), if appropriate. People who were about to undergo major surgery or a significant change to their medication, those with significantly impaired cognitive functioning or psychosis, inability to speak English. **Diagnosis of vestibular migraine:** The article states that a diagnosis of definite or probable vestibular migraine was made according to the criteria of Neuhauser 2001	
Interventions	**Intervention (n = 33 randomised, n = 16 completed)** A CBT programme designed to be delivered across 8 weeks in 2‐hour long closed group sessions (groups of 4 to 7 participants) was offered. The primary treatment components were: Education about vestibular migraine, including information about cause, symptoms, prognosis, medication and lifestyle Behavioural evidence including in‐session experiments and planned home‐based exposure tasks helped participants to test their perceived limits and accompanying beliefs about the utility of avoidance and (unnecessary) safety precautions as coping mechanisms Cognitive restructuring helped participants to identify their unhelpful thoughts and feelings about their VM and related stressors. These sessions introduced ways to challenge unhelpful beliefs and their effect on participants’ lifestyle. Skills focused on relapse prevention were also included. Each session was accompanied by a standard set of home‐based tasks to be completed before the next session. Three therapists were involved in the delivery of the programme: 2 clinical psychologists and 1 psychologist; and the groups were co‐facilitated by intern psychologists **Comparator (n = 28 randomised, n = 18 completed)** Wait‐list control. Participants were offered no intervention. At the end of the study, participants were able to receive CBT, as above. **Background interventions administered to all participants** Participants also received standard medical care for vestibular migraine. No specific interventions were offered as part of this study.	
Outcomes	**Primary outcomes relevant to this review:** **Improvement in vertigo** Not reported **Change in vertigo** Assessed using the VSS (Vertigo Symptom Scale short form). Assumed to be assessed at the end of treatment (8 weeks). Range 0 to 60, higher scores represent worse symptoms, and a score of ≥ 12 indicates severe dizziness. Subscales for vertigo/balance questions were not reported. **Serious adverse events** Not fully assessed or reported. Quote: "Although the treatment intervention we tested has been shown to be acceptable and effective for those who completed it we cannot deny the possibility that individuals within the sample may had had adverse responses." **Secondary outcomes relevant to this review:** **Disease‐specific health‐related quality of life** Assessed using the DHI at the end of treatment (8 weeks). Assessed using the Migraine Disability Assessment Scale (MIDAS) This includes 5 questions, scoring the number of days over the past 3 months that one's work and non‐work activity was reduced as a result of headache. Score ranges from 0 to 270. Scores 0 to 5: little or no disability; score 6 to 10: mild disability; score 11 to 20 moderate disability; score ≥ 21 severe disability. We note that not all participants suffered with headache. **Improvement in headache** Not reported **Improvement in other migrainous symptoms ** Not reported **Other adverse effects** Not fully assessed or reported. Quote: "Although the treatment intervention we tested has been shown to be acceptable and effective for those who completed it we cannot deny the possibility that individuals within the sample may had had adverse responses" **Other outcomes reported in the study:** Hospital Anxiety and Depression Scale WHOQoL (generic quality of life measure)	
Notes	**Research integrity checklist:** No retractions or expressions of concern were identified Baseline characteristics of the groups are not fully reported Plausible dropout was reported The study is free from any implausible results The numbers randomised to each group suggest that adequate randomisation was used	
***Risk of bias***		
**Bias**	**Authors' judgement**	**Support for judgement**
Random sequence generation (selection bias)	Low risk	Quote: "Simple randomisation using a randomisation table created by computer software (i.e. computerised sequence generation)."
Allocation concealment (selection bias)	Low risk	Quote: "After completing the assessment, participants were then randomly allocated on the basis of a computer generated random sequence (simple randomisation), to the waitlist group or the CBT group. A random set of numbers was generated by a researcher not involved in the assessment of the participants and linked in advance to a set of participant numbers. These allocations were concealed from the researcher until the participant completed the assessment."
Blinding of participants and personnel (performance bias) All outcomes	High risk	Quote: "Due to the nature of the study and the intervention, the lead researcher and therapists were unable to be blind to the allocation of participants."
Blinding of outcome assessment (detection bias) All outcomes	High risk	Comment: outcomes were reported by (unblinded) participants.
Incomplete outcome data (attrition bias) All outcomes	High risk	Comment: considerable dropout over the course of the trial for both groups, sufficient to affect the results.
Selective reporting (reporting bias)	Low risk	Comment: outcomes reported as pre‐specified in the trial protocol.
Other bias	Low risk	Comment: no other concerns.

**Table:** Qi 2020

***Study characteristics***		
Methods	Placebo‐controlled, parallel‐group, double‐blind RCT with 4 months duration of treatment and follow‐up	
Participants	**Setting: ** Single‐centre trial conducted at Cangzhou Central Hospital, China **Sample size:** **Number randomised: ** 218 participants **Number completed: ** 204 participants **Participant baseline characteristics** **Age: ** Probiotic group: mean 32 years (range 18 to 50) Control group: mean 33 years (range 19 to 49) **Gender: ** Probiotic group: 89 females (86.4%): 14 males (13.6%) Control group: 85 females (84.2%): 16 males (15.8%) **Probable/definite vestibular migraine: ** All participants had definite vestibular migraine **Attack frequency at baseline: ** Probiotic group: median 2.1 attacks per week (IQR 0.8 to 3.2) Control group: median 1.9 attacks per week (IQR 0.7 to 3.1) **Duration of disease:** Not reported **Inclusion criteria:** Aged 18 to 50 years old. Provided written, informed consent. Sufficient cognitive abilities as well as language proficiency to complete the assessments and questionnaires. Vestibular migraine (according to diagnostic criteria listed below). **Exclusion criteria:** Bilateral vestibular dysfunction. Report of mere spontaneous episodic dizziness that was not provoked/worsened by movements. Previous history of moderate neurological or orthopaedic deficits. Use of probiotics supplement within 2 months prior to this study. **Diagnosis of vestibular migraine:** IHS and Bárány Society criteria for definite vestibular migraine	
Interventions	**Intervention (n = 109 randomised, n = 103 completed)** Patients were prescribed with a daily dose of 1 capsule containing 2 × 10^10^ colony forming units of *Lactobacillus casei Shirota*, for a period of 4 months **Comparator (n = 109 randomised, n = 101 completed)** Patients in the placebo group were prescribed with a daily dose of 1 capsule containing corn starch (as placebo), also for a period of 4 months **Background interventions administered to all participants** Patients were asked to refrain from consuming any other probiotics supplement during the 4‐month study period, except for those prescribed to them through this study	
Outcomes	**Primary outcomes relevant to this review:** **Improvement in vertigo** No dichotomous data were reported, despite the methods section of this study indicating that a reduction in attack number would be analysed as categorical data. "The reduction in the attack number was categorized as complete resolution, substantial control (>50% decrease), moderate control (25‐50% decrease), and minimal control (<25% decrease) with unaltered or increased frequency". These data were not reported in the results. **Change in vertigo** This was assessed using a vertigo severity score, on a 10‐point Likert scale. Lower scores represent an improvement in vertigo symptoms. this scale may capture symptom severity as well as quality of life: "[...] which reflects the seriousness of vertiginous attack that negatively affects the life quality of the patients" It was also assessed with the frequency of vertigo episodes (number of attacks per week). **Serious adverse events** Not fully reported. The authors state "No side or adverse effects were observed in any patients during the entire study period." It is unclear if these were systematically assessed and reported. **Secondary outcomes relevant to this review:** **Disease‐specific health‐related quality of life** Assessed with the DHI at 2 months and 4 months of follow‐up **Improvement in headache** Not reported **Improvement in other migrainous symptoms ** Not reported **Other adverse effects** Not fully reported. The authors state "No side or adverse effects were observed in any patients during the entire study period." It is unclear if these were systematically assessed and reported. **Other outcomes reported in the study:** Beck Anxiety Inventory Beck Depression Inventory	
Notes	**Research Integrity Checklist:** No retractions or expressions of concern were identified The trial was registered with ChiCTR Database (ChiCTR2000033771) but we note that there is a discrepancy in the dates reported for the trial, which is suggestive that registration was retrospective The baseline characteristics of participants were not excessively similar Plausible dropout is reported No implausible results were noted Identical numbers were randomised to each group, but blocked randomisation was used	
***Risk of bias***		
**Bias**	**Authors' judgement**	**Support for judgement**
Random sequence generation (selection bias)	Unclear risk	Quote: "Eligible patients were assigned to either LcS or placebo group, using a random permutated block stratified to their baseline total Dizziness Handicap Inventory (DHI) scores with a size of 6 generated by a lab technician not participating in our current study". Comment: no information on generation of random sequence.
Allocation concealment (selection bias)	Low risk	Quote: "eligible patients were assigned [...] using a random permuted block [...] generated by a lab technician [..,] and distributed in sealed envelopes by a nurse not participating in the study." Comment: apparently adequate concealment of allocation.
Blinding of participants and personnel (performance bias) All outcomes	Low risk	Quote: "Data recording of endpoints were performed by an investigator who was blind to the randomisation process. The random numbers specifying patient group assignment were only revealed to investigators after the whole study was completed". Comment: participants and personnel appear to have been blinded to group allocation.
Blinding of outcome assessment (detection bias) All outcomes	Low risk	Comment: all outcomes were reported by blinded participants.
Incomplete outcome data (attrition bias) All outcomes	Low risk	Comment: number of dropouts was balanced across the groups, and fewer than 10% of participants. Probably unlikely to cause a large difference in the estimated effect size.
Selective reporting (reporting bias)	High risk	Methods state that vertigo was assessed as follows: "The reduction in the attack number was categorized as complete resolution, substantial control (>50% decrease), moderate control (25‐50% decrease), and minimal control (<25% decrease) with unaltered or increased frequency". However, this outcome is reported on a continuous scale (number of attacks per week) and is not reported as "improvement" as stated in the methods. No published protocol has been identified.
Other bias	Unclear risk	Quote: "the study spanned a period of two years, during which the patients were prescribed with various type and doses of medication by several doctors according to their condition at that time". Comment: medications used by participants at baseline and throughout the trial are not reported. Assessment of vertigo severity may have been conducted with an unvalidated scale. Concern over discrepancy in trial reported dates. Authors state that a per protocol analysis was conducted, although the majority of participants provided outcome data, and no details are provided regarding the number of participants who did not receive the intervention as planned.

CBT: cognitive behavioural therapy; DHI: Dizziness Handicap Inventory; ICHD International Classification of Headache Disorders; IHS: International Headache Society; IQR inter‐quartile range; MIDAS: Migraine Disability Assessment Scale; RCT: randomised controlled trial; SD: standard deviation; VM: vestibular migraine

### Characteristics of excluded studies [ordered by study ID]

**Table:**

Study	Reason for exclusion
ACTRN12616000683437	This study is not an RCT
Balci 2022	This is not an RCT; it was an observational cohort study with a pre‐post study design
Byun 2021	This is a systematic review and meta‐analysis. The reference list has been checked to ensure that any relevant studies have been included in this review.
ChiCTR1800014766	This study is not an RCT
CTRI/2022/01/039831	This ongoing RCT has an incorrect comparator for inclusion in this review. The control group received an active intervention (head and neck motion exercises).
Hu 2021	This ongoing study will compare acupuncture to venlafaxine (rather than no treatment or placebo), therefore the comparator is not relevant for this review
Koc 2021	This study is not an RCT. Participants with vestibular migraine were compared to those with other causes of vestibular dysfunction.
Liu 2013	The treatment used in this study comprised multiple interventions: herbal medications plus massage, followed by physical therapy and traction, therefore was not relevant for this review
NCT03979677	This study is not an RCT
NCT05508139	This is an observational cohort study, not an RCT
Sun 2022	The intervention in this study (resistance exercise) was compared to a group who received relaxation therapy, rather than no intervention or sham treatment. Therefore the comparator is not relevant for this review.
Zhang 2012	The intervention is a traditional Chinese medicine, therefore is not relevant for this review

RCT: randomised controlled trial

### Characteristics of ongoing studies [ordered by study ID]

**Table:** ChiCTR2000037202

Study name	'A randomized controlled study for Yunping Recipe in the treatment of vestibular migraine with syndrome of hyperactivity of liver yang'
Methods	Parallel‐group randomised controlled trial
Participants	Adults (aged 18 to 80 years) with a diagnosis of vestibular migraine
Interventions	Participants will receive Yunping recipe or a placebo. We have attempted to contact the authors to clarify what this intervention comprises, but have received no reply.
Outcomes	Dizziness Handicap Inventory (measured at 4, 8 and 12 weeks) Blood tests (liver and kidney function) and electrocardiogram
Starting date	Study dates stated as 1 December 2020 to 1 December 2022. However, the trial registry site still lists this study as "not yet recruiting".
Contact information	Zhang Lingling Email: yinghytz@126.com
Notes	It is possible that Yunping Recipe is a traditional Chinese medicine, therefore this trial may not be relevant for future iterations of this review

**Table:** CTRI/2021/12/038948

Study name	'Effect of vestibular rehabilitation and relaxation techniques in patients with migraine'
Methods	The study design is stated to be a parallel‐group randomised controlled trial
Participants	The inclusion criteria state that participants must have a diagnosis of migraine headache, and be aged over 18 years. It is not clear from the trial description whether participants will have vestibular migraine. However, due to the nature of the intervention, we have considered that this may be a relevant study for future iterations of this review.
Interventions	Vestibular rehabilitation and relaxation techniques, 6 sessions, conducted over a 2‐week period
Outcomes	Vertigo symptom scale Migraine specific quality of life
Starting date	30 December 2021 We note that the trial registry site still states "Not yet recruiting" (accessed 22 November 2022)
Contact information	Anushka Saboo sabooanushka97@gmail.com
Notes	As noted above, it is unclear whether the population included in this study is appropriate. Further details will need to be identified when the study is published.

## Differences between protocol and review

We planned to use the Trustworthiness Screening Tool from the Cochrane Pregnancy and Childbirth Group to identify studies for inclusion in the main analysis (Webster 2022c). However, as described in Selection of studies, we did not exclude studies from the main analysis on the basis of concerns whilst using this tool. We considered that the overall certainty of the review findings (all very low‐ or low‐certainty) would not be impacted by this decision. 
